# Antiviral responses are shaped by heterogeneity in viral replication dynamics

**DOI:** 10.1038/s41564-023-01501-z

**Published:** 2023-10-09

**Authors:** Lucas J. M. Bruurs, Micha Müller, Jelle G. Schipper, Huib H. Rabouw, Sanne Boersma, Frank J. M. van Kuppeveld, Marvin E. Tanenbaum

**Affiliations:** 1 Oncode Institute, Hubrecht Institute–KNAW and University Medical Center Utrecht, Utrecht, the Netherlands; 2 Virology Division, Department of Infectious Diseases and Immunology, Faculty of Veterinary Medicine, Utrecht University, Utrecht, the Netherlands; 3 Department of Bionanoscience, Delft University of Technology, Delft, the Netherlands

**Keywords:** RIG-I-like receptors, Virus-host interactions, Viral host response

## Abstract

Antiviral signalling, which can be activated in host cells upon virus infection, restricts virus replication and communicates infection status to neighbouring cells. The antiviral response is heterogeneous, both quantitatively (efficiency of response activation) and qualitatively (transcribed antiviral gene set). To investigate the basis of this heterogeneity, we combined Virus Infection Real-time IMaging (VIRIM), a live-cell single-molecule imaging method, with real-time readouts of the dsRNA sensing pathway to analyse the response of human cells to encephalomyocarditis virus (EMCV) infection. We find that cell-to-cell heterogeneity in viral replication rates early in infection affect the efficiency of antiviral response activation, with lower replication rates leading to more antiviral response activation. Furthermore, we show that qualitatively distinct antiviral responses can be linked to the strength of the antiviral signalling pathway. Our analyses identify variation in early viral replication rates as an important parameter contributing to heterogeneity in antiviral response activation.

## Abstract (web summary)

Variation in infection dynamics and host cell sensing kinetics shape human antiviral response.

## Main

The innate immune system provides a first line of defence against viral infection and stimulates activation of the adaptive immune system^1,2^. One step in innate immune activation is the production of type I interferons (IFN), which are important signalling molecules that induce an antiviral state in neighbouring cells and thereby protect these cells against viral infection^2,3^. However, excessive activation of IFN signalling can be toxic to tissues and contribute to hyperinflammation, which can contribute to various pathologies including coronavirus disease 2019 (COVID-19) (refs. ^4–7^). In addition, in the absence of infection, stringent control of antiviral response activation is required to prevent a spurious response, which can cause ‘interferonopathy’ syndromes^8,9^.

The antiviral response is triggered by detection of viral infection in the host cell^1^. For RNA viruses, double-stranded RNA (dsRNA) is an important ligand for activating the cellular antiviral response^10^. RIG-I-like receptors (RLRs), including melanoma differentiation-associated protein 5 (MDA5), can sense cytosolic dsRNA^10–12^. Binding of MDA5 to dsRNA activates a signalling pathway that culminates in nuclear translocation of the interferon regulatory factor (IRF) family of transcription factors^1^. Nuclear IRFs induce transcription of several genes with antiviral functions (for example, *IFIT1* and *RSAD2*) as well as proinflammatory cytokines, including *IFNs*, for which the protein products are secreted and can induce expression of antiviral interferon stimulated genes (ISGs) in uninfected neighbouring cells^2,13–17^.

To prevent an antiviral response by the host cell, viruses have evolved strategies to repress activation of the IFN pathway and to inhibit expression of antiviral genes^18–20^. Nevertheless, a subset of infected cells is capable of launching an antiviral response and expressing IRF target genes, resulting in cell-to-cell heterogeneity in the antiviral response^20^. For example, infections with different viruses result in *IFNB1* expression in <1%–30% of infected cells^21–25^. The antiviral response can also differ qualitatively, even among neighbouring infected cells; the set of transcribed antiviral genes can vary between infected cells of the same cell type^21,23,26^, creating an additional layer of cell-to-cell heterogeneity in the antiviral response.

Although heterogeneity in the antiviral response has been reported and probably has a role in controlling viral spread, it is poorly understood. Exogenous overexpression of host cell proteins of the dsRNA sensing pathway (for example, MDA5, TBK1, MAVS, IRF3) increased the fraction of *IFNB1-*producing cells^21,22^, suggesting that these proteins affect the efficiency of antiviral response activation. However, it is unclear whether endogenous expression of these proteins varies between cells and whether the extent of such variation is sufficient to explain the observed heterogeneity in antiviral response activation^27^. Cell-to-cell heterogeneity in the antiviral response has also been reported in sister cells after cell division, suggesting that factors other than host gene expression differences might affect heterogeneity in the antiviral response^28,29^.

Considerable variation also exists in the progression of viral infection. For example, viral replication rates vary among infected cells, possibly as a result of differences in the infecting virus (for example, variations in viral genome sequences) or through differences in the host cell (for example, expression levels of factors that aid virus replication)^30–33^. Since viral replication rates and antiviral responses show cell-to-cell heterogeneity, it is possible that heterogeneity in viral replication is causally linked to heterogeneity in antiviral signalling. However, some studies have reported a positive correlation between viral load and antiviral signalling^22,34^, while others reported either negative^23,35^ or no correlation^21,24^. Most studies so far have used single-timepoint measurements, for example, quantitative PCR, fluorescence in situ hybridization (FISH) or RNA-sequencing to determine viral genome abundance. A major limitation of such measurements is that they cannot take into account variability in the start of infection in different cells. Assessing the moment of infection is crucial to discriminate cells with low viral replication rates from cells in which infection initiated later in the experiment. Moreover, several studies used infections with a multiplicity of infection (MOI) > 1 (refs. ^21,22,25^), resulting in considerable variation in the number of virions that infect a single cell. Because MOI affects the rate of virus replication^32,36^, it is challenging to disentangle heterogeneity in viral replication rates from variation in the number of infecting virions in infections with MOI > 1. To study the effect of viral replication rates on innate immune activation, highly sensitive live-cell readouts are required to precisely determine the moment of infection by individual viruses as well as the timing and strength of antiviral response activation in single cells.

Here we combine Virus Infection Real-time IMaging (VIRIM), a live-cell single-molecule imaging method for detecting viral infection and replication^37^, with real-time, highly sensitive readouts of the dsRNA sensing pathway and antiviral response activation to assess whether variation in viral replication rates contributes to heterogeneity in antiviral response activation.

## Results

### Real-time imaging of encephalomyocarditis virus (EMCV) infection

**Fig. 1:**
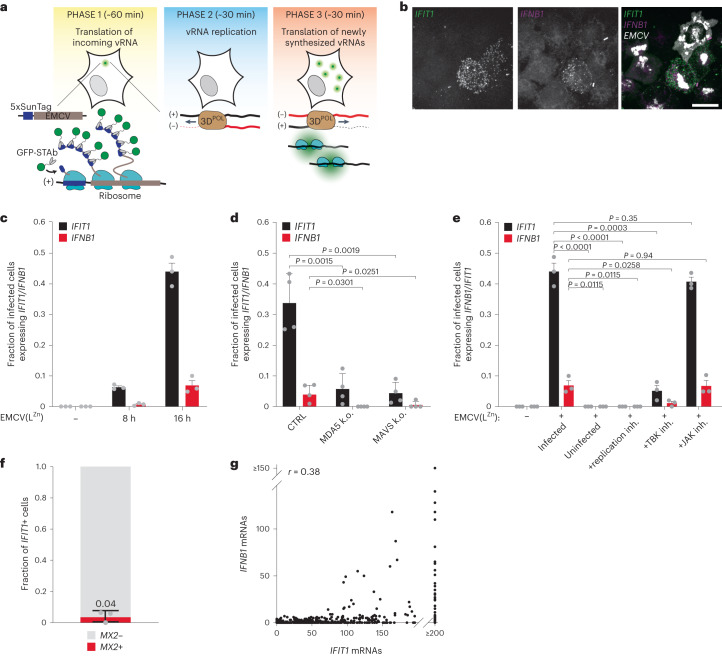
IFIT1 is expressed in an MDA5/MAVS/TBK-dependent manner in EMCV(L^Zn^)-infected cells. **a**, Scheme of VIRIM experimental setup and VIRIM phases. During phase 1, a single GFP spot is visible, which represents the translated incoming vRNA. In phase 2, translation of the incoming vRNA is terminated and the vRNA undergoes replication, resulting in the disappearance of the GFP spot. In phase 3, newly synthesized vRNAs are produced and translated, resulting in the appearance of new GFP spots. Average phase durations are provided in minutes. 3D^POL^, RNA-dependent RNA polymerase. **b**, Representative smFISH image of 5xSunTag-EMCV(L^Zn^)-infected HeLa cells at 16 h.p.i., labelled with probes targeting *IFIT1* and *IFNB1* mRNAs and viral EMCV genomes. Scale bar, 20 µm. **c**, Fraction of (infected) cells with >10 *IFNB1* (red bars) or >20 *IFIT1* (black bars) mRNAs in uninfected cells and cells at 8 and 16 h.p.i. (*n* = 3 independent experiments). **d**, Fraction of infected cells expressing >10 *IFNB1* or >20 *IFIT1* mRNAs at 16 h.p.i. in either HeLa control or MDA5 and MAVS k.o. HeLa cells (*n* = 4 independent experiments). **e**, Fraction of infected cells expressing >10 *IFNB1* or >20 *IFIT1* mRNAs at 16 h.p.i. with or without treatment with an EMCV replication inhibitor (DiP), TBK1/IKKε inhibitor (MRT) or JAK1/3 inhibitor (TOFA) (*n* = 3 independent experiments). **f**, Fraction of *IFIT1+* cells expressing >5 *MX2* mRNAs at 16 h.p.i. A value of 0.04 indicates that on average, 4% of *IFIT1+* cells are positive for *MX2* expression (*n* = 3 independent experiments). **g**, Scatterplot showing the number of *IFIT1* and *IFNB1* mRNAs in 5xSunTag-EMCV(L^Zn^) infected cells 16 h.p.i. (*r* indicates Pearson’s correlation coefficient, *n* = 771 cells, 3 independent experiments). Grey dots in **c**–**f** represent values of individual biological replicates. All bars and error bars indicate mean ± s.e.m. *P* values in **d** and **e** were determined using two-sided, paired-samples *t*-test.

EMCV is a picornavirus model to study antiviral responses^19,38–41^. We previously reported a live-cell imaging assay for EMCV named VIRIM^37^ that uses two components to visualize virus infection: first, 5 copies of a short peptide called the SunTag^42^ are inserted at the N terminus of the viral polyprotein (5xSunTag-EMCV). The second component of VIRIM consists of a genetically encoded single-chain variable fragment (scFv) antibody that binds tightly to the SunTag peptide (referred to as SunTag antibody, STAb) and is fused to a green fluorescent protein (GFP-STAb). When the 5xSunTag-EMCV genome is translated in GFP-STAb-expressing cells, SunTag peptides are co-translationally bound by the GFP-STAb (Fig. 1a). Since each viral RNA (vRNA) is translated by multiple ribosomes, many GFP-STAb molecules are recruited to a single translating vRNA, resulting in a bright fluorescent spot that can be detected by spinning disc confocal microscopy^37^. The number of fluorescent SunTag GFP foci in a cell accurately reports on the number of viral genomes and can therefore be used both to determine the start of vRNA translation and to assess viral replication kinetics early in infection^37^.

**Extended Data Fig. 1:**
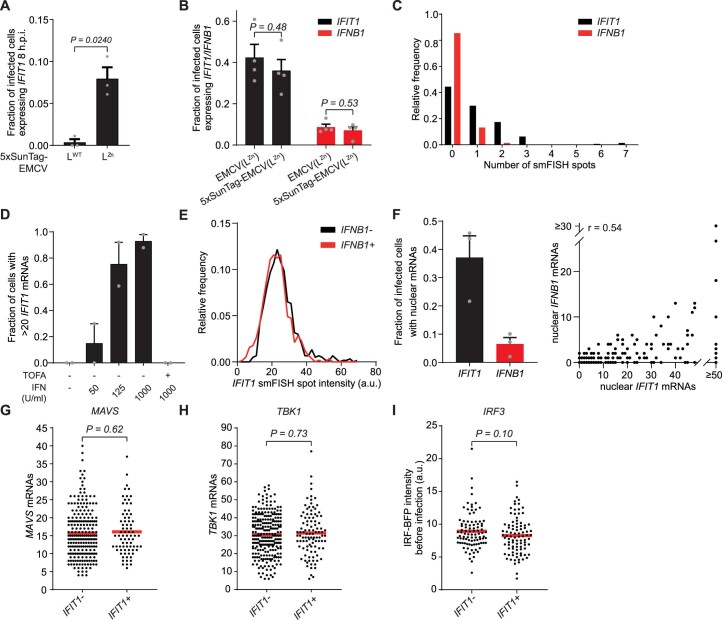
Extended data related to figure 1. **a**) Fraction of 5xSunTag-EMCV(L^WT^) or 5xSunTag-EMCV(L^Zn^) infected cells expressing 20 or more *IFIT1* mRNAs at 8 h.p.i. (n = 3 independent experiments) *P* value determined using two-sided, independent samples T-test. **b**) Fraction of EMCV(L^Zn^) or 5xSunTag-EMCV(L^Zn^) infected cells expressing >20 *IFIT1* or >10 *IFNB1* mRNAs at 16 h.p.i. (n = 577 and 532 cells for EMCV(L^Zn^) and 5xSunTag EMCV(L^Zn^) respectively, 4 independent experiments) *P* value determined using two-sided, independent samples T-test. **c**) Histogram of the number of *IFIT1* (black bars) and *IFNB1* (red bars) smFISH spots in cells which were not incubated with virus (n = 144 cells, 3 independent experiments). **d**) Fraction of cells that express more than 20 *IFIT1* mRNAs in cells that were treated with different concentrations of IFN for 24 h in the presence or absence of the the JAK1/3 inhibitor Tofacitinib (TOFA.) (n = 2 independent experiments). **e**) *IFIT1* smFISH spot intensity distribution in *IFNB1-* (black line) and *IFNB1* + (red line) cells (n = 600 and 570 spots respectively, 3 independent experiments). **f**) Left panel: Fraction of infected cells that have >10 *IFIT1* and > 5*IFNB1* nuclear mRNAs at 16 h.p.i. Right panel: Scatter plot showing the number of *IFIT1* and *IFNB1* mRNAs in the nucleus in 5xSunTag-EMCV(L^Zn^) infected cells 16 h.p.i. (r indicates Pearson’s correlation coefficient, n = 269 cells, 3 independent experiments). **g, h**) *MAVS* (G) and *TBK1* (H) mRNA expression levels at 8 h.p.i. in *IFIT1-* or *IFIT1+* cells. Spots represent single cells and red lines indicate averages (MAVS: n = 222 and 77, TBK1: n = 238 and 103 in IFIT1- and IFIT1+ infections respectively, 3 independent experiments). *P* values were determined using two-sided, Mann-Whitney test. **i**) IRF3-BFP expression levels at the start of infection in cells that become either *IFIT1-* or *IFIT1+* at 16 h.p.i. Spots represent single cells and red lines indicate averages (n = 93 and 92 *IFIT1-* and *IFIT1+* infections respectively, 6 independent experiments). *P* value determined using two-sided, Mann-Whitney test. Grey dots in A,B,D,F represent in individual biological replicates. Bars and error bars indicate average ± s.e.m. in all panels.

EMCV infection results in potent inhibition of the dsRNA sensing pathway, thereby preventing expression of IRF3 target genes^41^, and limiting our ability to study viral sensing and activation of the antiviral response pathway. Although several EMCV proteins are implicated in suppressing the dsRNA sensing pathway, the Leader (L) protein is considered the main IFN antagonist of EMCV^41,43–45^. Indeed, an EMCV virus with inactivating mutations in the zinc finger domain of the L protein (‘EMCV(L^Zn^)’) induces potent expression of antiviral genes^41^, which we confirmed by single-molecule (sm)FISH (Extended Data Fig. 1a). Importantly, we find that EMCV(L^Zn^) infection induced antiviral gene expression only in a subset of infected cells, indicating that L protein inactivation does not result in a loss of cell-to-cell heterogeneity in antiviral response activation (Fig. 1b and Extended Data Fig. 1a). Furthermore, introduction of the 5xSunTag array into the viral genome did not affect the efficiency of antiviral response activation (Extended Data Fig. 1b).

### Single-cell analyses of IFIT1 and IFNB1 expression

To monitor activation of the antiviral response, we searched for genes that are transcriptionally activated in cells that have sensed viral dsRNA via the MDA5/MAVS/IRF pathway. *IFNB1*, the best known IRF3 target gene, is only expressed in a subset of cells in which IRF3 is activated^21^ and is therefore not a suitable marker gene. We chose the gene encoding interferon induced protein with tetratricopeptide repeats 1 (*IFIT1*). *IFIT1* is best known as an ISG, but its expression is also upregulated by IRF3-dependent transcription in an IFN-independent manner^17,46,47^. *IFIT1* transcription is strongly induced during virus infection and induction of *IFIT1* expression can be detected well before *IFNB1* expression^21^. We assessed *IFIT1* and *IFNB1* expression in HeLa cells in response to 5xSunTag-EMCV(L^Zn^) infection using smFISH—a sensitive, single-cell method for analysis of gene expression. smFISH probes targeting *IFIT1* and *IFNB1* mRNAs were combined with probes targeting the EMCV genome to identify infected cells (Fig. 1b). Baseline expression of *IFIT1* and *IFNB1* in uninfected HeLa cells is very low (98% of uninfected cells have <4 *IFIT1* and <2 *IFNB1* mRNAs) (Extended Data Fig. 1c). *IFIT1* and *IFNB1* expression was induced by viral infection, and we found that the fraction of cells expressing *IFIT1* was higher than the fraction expressing *IFNB1* at multiple timepoints in infection (Fig. 1c). These data support using *IFIT1* expression as a readout for activation of antiviral signalling. Notably, even at the final timepoint (16 hours post infection (h.p.i.)), after which we observed cell death of EMCV-infected cells, less than half of infected cells showed *IFIT1* expression, indicating heterogeneity of the antiviral response following 5xSunTag-EMCV(L^Zn^) infection.

Next, we confirmed that expression of *IFIT1* (and *IFNB1*) is induced by detection of viral dsRNA in the infected host cell, rather than by paracrine IFN signalling. First, we found that deletion of the dsRNA sensor MDA5 or downstream inactivation of the dsRNA sensing pathway, either by using MAVS knockout (k.o.) cells or pharmacological inhibition of TBK1 (the kinase responsible for IRF3 activation), resulted in a strong reduction of *IFIT1* and *IFNB1* expression (Fig. 1d,e). In contrast, inhibition of paracrine IFN signalling through JAK inhibition did not affect expression of *IFIT1* and *IFNB1* in response to EMCV infection (Fig. 1e and Extended Data Fig. 1d). Second, *IFIT1* and *IFNB1* expression required viral replication, as inhibition of EMCV replication by dipyridamole (DiP)^48^ results in complete loss of their expression (Fig. 1e). Third, the vast majority (96%) of *IFIT1*-positive cells is negative for expression of *MX2*, a typical ISG that is expressed in response to IFN paracrine signalling (Fig. 1f)^49^. Lastly, expression of *IFIT1* is limited to infected cells and is not observed in uninfected neighbouring cells (Fig. 1e, ‘uninfected’). Thus, even though *IFNB1* transcripts are observed in a small fraction of infected cells, this does not lead to notable paracrine *IFIT1* induction, possibly because insufficient IFN is produced under our experimental conditions to induce paracrine signalling, or because IFN protein is not efficiently produced due to translation inhibition in EMCV-infected cells. We also included analysis of *IFIT1* expression in uninfected neighbouring cells in subsequent experiments to confirm the absence of paracrine *IFIT1* activation in each experiment. Together, these experiments establish *IFIT1* expression as a sensitive marker for cells that sense intracellular infection through viral dsRNA and activate an antiviral response.

Simultaneous smFISH labelling of *IFIT1* and *IFNB1* mRNAs in single cells showed that all *IFNB1+* cells express *IFIT1*, but that not all *IFIT1+* cells express *IFNB1* (Fig. 1g). This confirms that *IFNB1+* cells are a subset of *IFIT1+* cells. We confirmed that the absence of *IFNB1* mRNAs in some *IFIT1+* cells is neither the result of poor smFISH labelling efficiency, nor of low cytosolic *IFNB1* mRNA stability (Extended Data Fig. 1e,f)^50^. Rather, differences in *IFIT1* and *IFNB1* expression originate during transcription. Interestingly, *IFNB1* expression is mainly observed in cells expressing high levels of *IFIT1* (Fig. 1g), suggesting that cells with very strong antiviral responses preferentially induce *IFNB1* transcription. Combining smFISH for *IFIT1* and *IFNB1* can reveal heterogeneity in host cell responses and allow identification of at least three quantitatively and qualitatively distinct host responses to viral infection: (1) no antiviral response activation (*IFIT1−/IFNB1*−), (2) activation of *IFIT1* expression only (*IFIT1*+*/IFNB1−*) and (3) activation of both *IFIT1* and *IFNB1* (*IFIT1*+*/IFNB1*+).

Heterogeneity in innate immune activation could arise from differences originating either in the virus or in the host. To test for host heterogeneity, we assessed endogenous expression levels of *MAVS*, *TBK1* and IRF3, as overexpression of these genes was previously reported to increase antiviral response activation^22^. We found no differences in expression of these genes in *IFIT1+* and *IFIT1*− cells (Extended Data Fig. 1g–i), suggesting that variation in expression of these proteins does not contribute to antiviral response heterogeneity. We also attempted to assess whether variation in *MDA5* expression causes heterogeneity in host response by smFISH but found that *MDA5* mRNA levels are altered during infection, precluding analysis of how *MDA5* levels before infection affect antiviral response activation. To circumvent this, we attempted to fluorescently label endogenous MDA5 protein but failed to detect fluorescence in single cells owing to low MDA5 expression levels.

### Viral load is lower in cells with active antiviral response

**Fig. 2:**
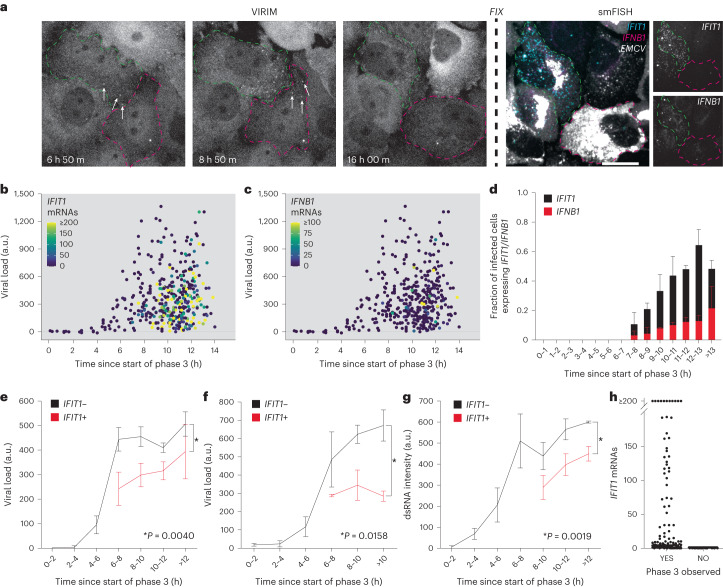
Antiviral response activated in cells with a lower viral load. **a**–**g**, For all panels, HeLa cells expressing GFP-STAb were infected with 5xSunTag-EMCV(L^Zn^) and imaged for 16 h. Then, cells were fixed and subjected to smFISH labelling using probes targeting *IFIT1* and either *IFNB1* mRNAs or EMCV genomes. In **g**, smFISH labelling was combined with immunofluorescence for dsRNA. **a**, Representative images of live-cell virus infection imaging using VIRIM combined with post-fixation smFISH for *IFIT1*, *IFNB1* and EMCV. Left (VIRIM): time since virus addition is noted. White arrows indicate GFP foci (translating vRNAs). Right: smFISH labelling of the infected cells with probes targeting *IFIT1* and *IFNB1* mRNA and EMCV genomes. Coloured dashed lines mark the outline of cell. Scale bar, 20 µm. **b**,**c**, Scatterplots showing viral load relative to the time in infection. Spot colour indicates number of *IFIT1* mRNAs (**b**) and *IFNB1* mRNAs (**c**) (*n* = 399 cells, 3 independent experiments). **d**, Fraction of infected cells expressing >20 *IFIT1* mRNAs (black bars) or >10 *IFNB1* mRNAs (red bars) at different time periods since the start of phase 3 (*n* = 399 cells, 3 independent experiments). **e**,**f**, Average viral load of 5xSunTag-EMCV(L^Zn^) (**e**) and 5xSunTag-EMCV(L^WT^) (**f**) infected *IFIT1*− and *IFIT1+* cells at different time periods in infection (**e**, *n* = 243 and 156 *IFIT1−* and *IFIT1+* cells, respectively, 3 independent experiments; **f**, *n* = 26 and 106 *IFIT1+* and *IFIT1*− cells, respectively, 6 independent experiments). **g**, Average dsRNA IF staining intensity of 5xSunTag-EMCV(L^Zn^) infected, *IFIT1−* and *IFIT1+* cells at different time periods in infection (relative to the start of phase 3) (*n* = 65 and 237 *IFIT1+* and *IFIT1−* cells, respectively, 3 independent experiments). **h**, Number of *IFIT1* mRNAs for cells in which infection did or did not progress to phase 3 (*n* = 118 and 24 phase 3+ and phase 3− cells, respectively, 3 independent experiments). In all panels, bars and error bars indicate mean ± s.e.m. *P* values in **e**–**g** were determined using two-way analysis of variance (ANOVA).

**Extended Data Fig. 2:**
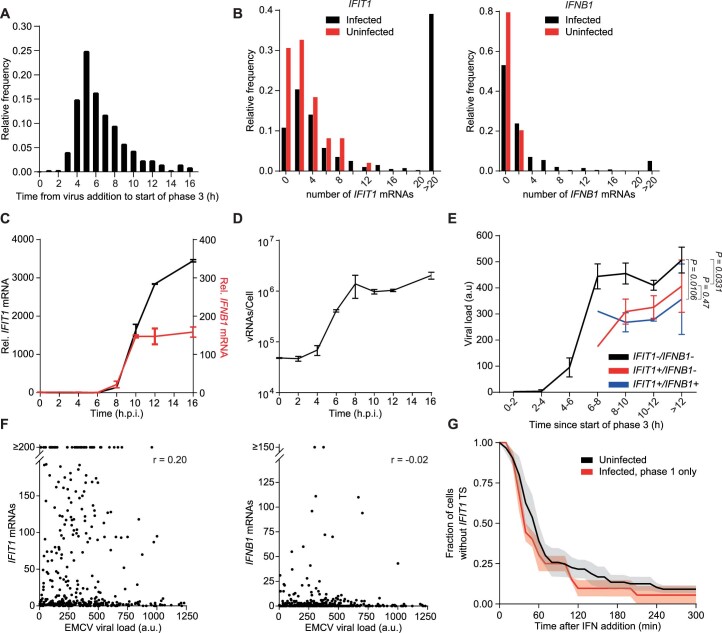
Extended data related to figure 2. **a**) Histogram of the time between the moment of virus addition to the cell culture medium and start of phase 3 (n = 399 infections, 3 independent experiments). **b**) Histogram of the number of *IFIT* (left) and *IFNB1* (right) mRNAs in infected (black bars) and uninfected, neighbouring (red bars) cells observed in the experiments of which the results are presented in Fig. 2b–e and h (n = 49 uninfected cells and 399 infected cells, 3 independent experiments). **c**) Relative *IFIT1* (black line, left y-axis) and *IFNB1* (red line, right y-axis) mRNA levels in HeLa cells expressing GFP-STAb infected with 5xSunTag-EMCV(L^Zn^) at different time points, as determined by qPCR. mRNA levels are expressed relative to expression at the moment of virus addition (t = 0) (n = 3 experiments). **d**) Viral genome abundance in HeLa cells expressing GFP-STAb infected with 5xSunTag-EMCV(L^Zn^) at different time points as determined by qPCR (n = 3 experiments). **e**) Average viral load of 5xSunTag-EMCV(L^Zn^) infected *IFIT1-/IFNB1-*, *IFIT1* + */IFNB1-*, and *IFIT1* + */IFNB1+* cells at different time periods since the start of phase 3 (n = 243, 122, and 34 cells, respectively, 3 independent experiments). *P* values were determined using a two-way ANOVA test. **f**) Scatter plot showing the viral load and either the number of *IFIT1* (left) or *IFNB1* (right) mRNAs at 16 h.p.i. (r indicates Pearson’s correlation coefficient, n = 399 cells, 3 independent experiments). **g**) Fraction of infected cells without *IFIT1* transcription site (TS) over time upon 1000U/ml IFN stimulation. Cells were infected and imaged for 16 h using VIRIM, after which cells were treated with IFN and IFIT1 transcription was imaged for 5 h. Appearance of an *IFIT1* TS was scored in uninfected cells black line) or in cells that experienced an abortive infection (‘Infected, phase 1 only’, red line). Line and light shading represent average and s.e.m. of 3 independent experiments (n = 67 uninfected cells and 20 abortive infections). Error bars indicate s.e.m. in all panels.

To test whether variation in viral replication dynamics can explain heterogeneity in antiviral response, we combined smFISH with live-cell VIRIM (Fig. 2a). Since not all infections initiate and progress simultaneously (Extended Data Fig. 2a), all infections were aligned in silico to the start of VIRIM infection phase 3 (which approximates the first moment in infection when viral dsRNA is produced, see Fig. 1a). This allowed us to determine how long each cell had been infected at the moment of fixation. This synchronization revealed that a considerable lag period (~7 h) exists between the first round of virus replication and the emergence of *IFIT1* and *IFNB1* transcripts (Fig. 2b–d and Extended Data Fig. 2b), which was confirmed by RT-qPCR (Extended Data Fig. 2c).

We determined the viral RNA load of cells at different timepoints in infection using FISH, allowing in silico reconstruction of vRNA increase over time during infection (Fig. 2e). This analysis revealed that the average vRNA load increased rapidly during the first 6–8 h and reached a plateau ~8 h after initiation of replication (that is, infection phase 3), comparable to what is observed when vRNA replication is measured by RT-qPCR (Fig. 2e, Extended Data Fig. 2d and Methods).

Interestingly, cells that activated an antiviral response, as determined by *IFIT1* expression, showed a lower average vRNA load than cells that do not express *IFIT1* (Fig. 2b,e). Similarly, the levels of viral dsRNA were lower in *IFIT1+* than in *IFIT1−* cells (Fig. 2g). Unfortunately, we were unable to establish whether *IFIT1*+*/IFNB1+* cells displayed a more prominent reduction in viral load due to the low number of such cells in the population (Extended Data Fig. 2e). Importantly, when cells were infected with 5xSunTag-EMCV(L^WT^) instead of 5xSunTag-EMCV(L^Zn^), we observed a similar correlation between viral load and *IFIT1* expression, indicating that even in the presence of potent suppression of antiviral signalling, activation of the antiviral response also negatively correlates with viral load (Fig. 2f). We did not detect any *IFNB1+* cells upon 5xSunTag-EMCV(L^WT^) infection, indicative of the potent antagonism exerted by the L protein and illustrating the benefit of using EMCV(L^Zn^) mutant virus and *IFIT1* as a reporter gene in studying antiviral responses. Notably, the ability to stratify cells using time-lapse microscopy data according to the duration of infection was crucial to reveal the correlation between viral load and innate immune response, as no correlation was observed when infection duration was not taken into account (Extended Data Fig. 2f).

Previously, using VIRIM on the related picornavirus CVB3, we found that ~20% of infections arrest before or during replication of the incoming vRNA (phase 2, Fig. 1a)^37^. Similarly, we found that ~15% of EMCV infections fail to progress beyond replication of the incoming vRNA. These abortive infections failed to induce *IFIT1* expression (Fig. 2h). This was not due to an inability of these cells to activate *IFIT1* transcription, as exogenous IFN stimulation resulted in potent *IFIT1* transcription in these cells (Extended Data Fig. 2g). One possible explanation for these findings is that infections that fail to complete replication of the incoming vRNA produce insufficient amounts of dsRNA for mounting an antiviral response.

### Viral replication rates affect antiviral response activation

**Extended Data Fig. 3:**
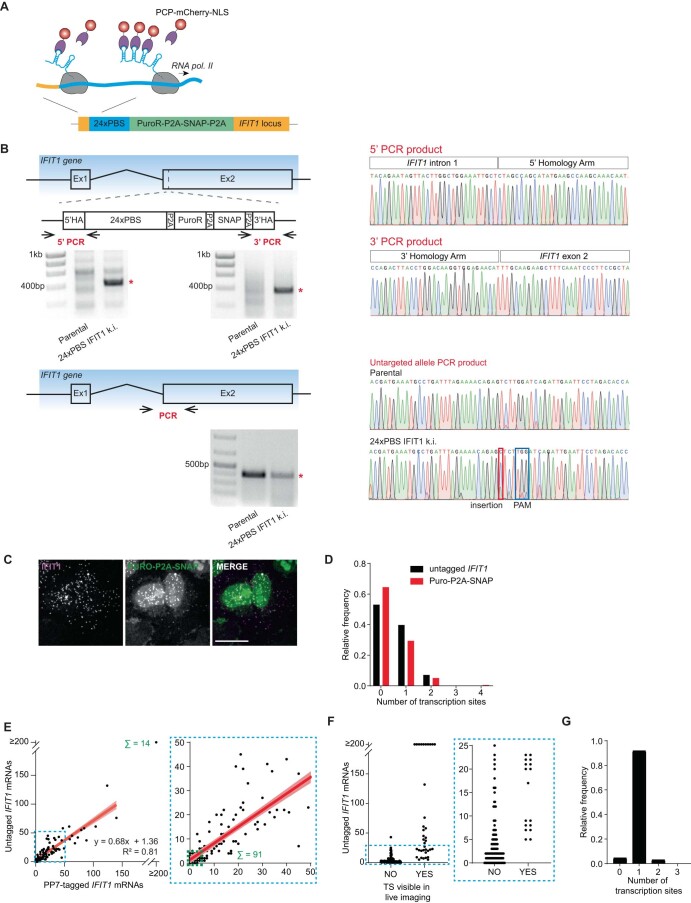
Validation of 24xPBS *IFIT1* reporter cell line. **a**) Schematic representation of *IFIT1* transcription imaging system using the PP7 system. PBS = PP7 binding site, PCP = PP7 coat protein. **b**) Schematic representation of the 24xPBS reporter gene integrated in the *IFIT1* gene locus and genotyping results. Left panels: PCR reactions confirm correct integration of the reporter (expected size of PCR fragment indicated with red asterisk). PCR fragments were also subjected to Sanger sequencing to confirm correct integration (right panels). Representative result of two repeats is shown. In the sequence trace the PAM sequence (blue box) and the location of a single nucleotide insertion (red box) are highlighted. For panels C-F, *24xPBS IFIT1* cells were infected with 5xSunTag-EMCV(L^Zn^) for 16 h and subjected to smFISH targeting the *IFIT1* coding sequence (labelling mRNAs derived from both the tagged and untagged *IFIT1* allele) and the Puro-P2A-SNAP coding sequence (labelling mRNAs derived from the tagged *IFIT1* allele only). **c**) Representative image of an infected cell subjected to dual smFISH labeling of PP7-tagged and untagged *IFIT1* mRNAs. Nuclear background signal in Puro-P2A-SNAP smFISH originates from residual PCP-mCherry-NLS fluorescence. Scale bar, 20 µm. **d**) Histogram of the number of untagged (black bars) or PP7-tagged (red bars) *IFIT1* transcription sites per cell (n = 196 cells, 4 independent experiments). **e**) Number of PP7-tagged and untagged *IFIT1* mRNAs in individual *24xPBS IFIT1* cells. Red line indicates linear regression of the data points with light red shading indicating the 95% confidence interval. Right scatter plot represents zoom in of the blue dashed box in the left graph (R^2^ indicates coefficient of determination, n = 177 cells, 3 independent experiments). Green Σ indicates the number of overlapping spots. **f**) Number of untagged *IFIT1* mRNAs in cells that did or did not develop an *IFIT1* transcription site during the 16 h of imaging. Right scatter plot represents zoom in of the blue dashed region in the left plot (n = 38 *IFIT1+* and 135 *IFIT1-* cells, 3 independent experiments). **g**) Number of *24xPBS IFIT1* transcription sites observed in 6 h of live imaging after IFNα2 stimulation (1000U/ml) (n = 494 cells, 3 independent experiments). Source data

Average vRNA levels in *IFIT1+* cells were lower than in *IFIT1*− cells. To distinguish whether viral replication rates determine the efficiency of antiviral response activation, or reduced vRNA loads in *IFIT1+* cells are the consequence of innate immune responses, we measured viral replication rates before innate immune activation had occurred; if viral replication rates are already lower in *IFIT1+* cells compared with *IFIT1−* cells before innate immune activation, this would indicate that the differences in vRNA load are not a consequence of innate immune activation, but rather would be consistent with slower viral replication causing increased innate immune activation. To assess innate immune activation in real time during infection, we generated a cell line to visualize *IFIT1* transcription with single mRNA sensitivity by integrating an array of 24 PP7 binding sites (PBS) into the endogenous *IFIT1* gene and expressing an mCherry-tagged PP7 coat protein (PCP), which binds with high affinity to the PBS (Methods)^51^. In this system, transcription of the 24xPBS-tagged *IFIT1* allele results in the appearance of a fluorescent spot at the site of transcription (Extended Data Fig. 3a). We confirmed that expression of the *24xPBS IFIT1* allele accurately reports on endogenous *IFIT1* transcription during EMCV infection and established that transcription imaging of the reporter allele provides a sensitive readout for antiviral response activation (Extended Data Fig. 3b–g and Methods). This real-time *IFIT1* transcription imaging system therefore allows live-cell analysis of innate immune activation and enables precise determination of the onset time of *IFIT1* transcription.

**Fig. 3:**
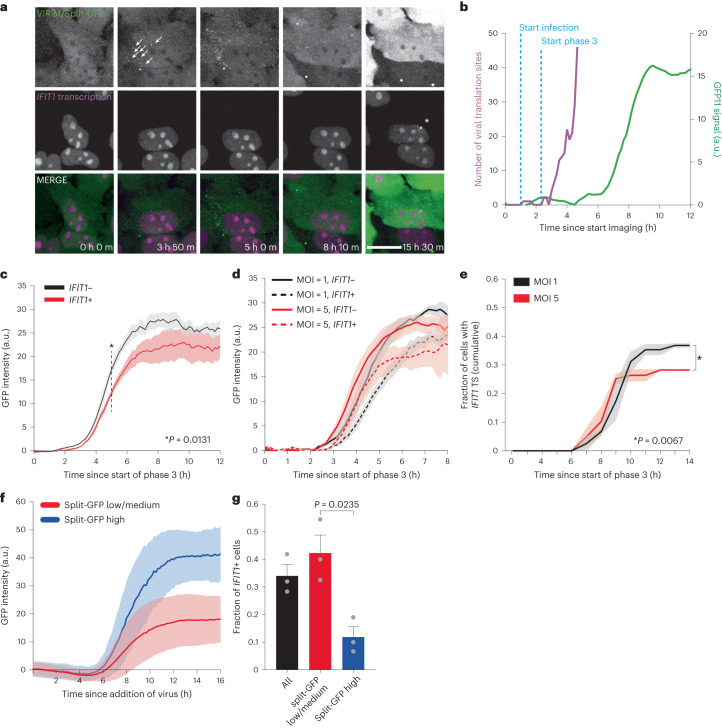
Early viral replication rates are slower in cells that activate an antiviral response. **a**–**g**, For all panels, *24xPBS* *IFIT1* k.i. cells expressing GFP-STAb, GFP(1–10) and PCP-mCherry-NLS were infected with GFP11-5xSunTag-EMCV(L^Zn^) and imaged for 16 h. **a**, Representative images from a 16 h time-lapse movie of cells infected with GFP11-5xSunTag-EMCV(L^Zn^). Top row: VIRIM (early timepoints) and split-GFP expression levels (late timepoints). White arrows at 3h50m timepoint highlight the appearance of newly translating vRNAs that mark the start of phase 3. Middle row: PCP-mCherry-NLS used for *IFIT1* transcription imaging. White asterisk indicates *IFIT1* transcription site. In some cells, cytosolic GFP aggregates can be observed, which result from GFP-STAb and GFP1–10 co-aggregation. Aggregates can easily be discriminated from VIRIM foci (Methods). Scale bar, 20 µm. **b**, Example intensity time traces of VIRIM foci number (purple line) and split-GFP signal (green line) in a GFP11-5xSunTag-EMCV(L^Zn^)-infected cell. **c**, Split-GFP signal accumulation in cells with (red line) and without (black line) *IFIT1* transcription. Line and light shading represent mean ± s.e.m. of 4 independent experiments (*n* = 46 *IFIT1+* and 117 *IFIT1*− infections). **d**, Split-GFP signal accumulation in *IFIT1*+ (dashed line) and *IFIT1*− (solid line) cells infected using either MOI = 1 (black lines) or MOI = 5 (red lines). Line and light shading represent mean ± s.e.m. of 3 independent experiments (*n* = 22 and 38 at MOI = 1 and *n* = 19 and 48 at MOI = 5 for *IFIT1+* and *IFIT1*− cells, respectively). **e**, Cumulative fraction of *IFIT1+* cells since the start of phase 3. Line and light shading indicate mean ± s.e.m. of 3 independent experiments (*n* = 60 (MOI = 1) and 67 (MOI = 5) infections). **f**, Split-GFP intensity time traces of split-GFP low/medium (red line) and high (blue line) infections. Line and light shading represent mean ± s.d. of 3 independent experiments (*n*: medium/low = 842, high = 129 infections). **g**, Average fraction of cells that activate *IFIT1* transcription in different infection clusters. Grey dots represent values from individual replicates (*n* = 3 experiments, error bars are s.e.m.). *P* values in **c**, **e** and **g** were determined using two-sided, paired-samples *t*-test at *t* = 5 h (dashed line in **c**) or 14 h (**e**).

While VIRIM allows sensitive quantitative measurements of viral replication during early infection, late-stage infection cannot be readily assessed because the large amounts of SunTag protein produced during later stages of infection ultimately sequesters all cellular GFP-STAb, resulting in decreased GFP-STAb labelling of translating viral genomes. To visualize both early and late infection in single cells, we made use of the split-GFP system, in which two non-fluorescent fragments of GFP (termed ‘GFP1–10’ and ‘GFP11’) bind each other, thereby reconstituting fluorescent GFP^52^. We generated GFP11-5xSunTag-EMCV virus and stably expressed the GFP1–10 fragment in the *24xPBS IFIT1* cell line expressing GFP-STAb, such that SunTag translation, split-GFP reconstitution and *IFIT1* transcription can all be visualized in the same cell (Fig. 3a and Supplementary Video 2). Importantly, the split-GFP system lacks the sensitivity of VIRIM during early infection, but reports on viral replication in later stages of infection and can be read out in the same cell (Fig. 3a,b and Methods). Thus, combining VIRIM with split-GFP imaging allows accurate determination of the start of infection and measurements of viral replication later in infection.

**Extended Data Fig. 4:**
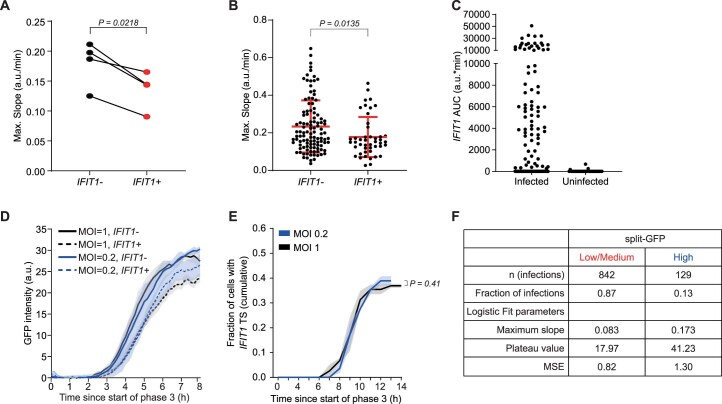
Extended data related to figure 3. **a**) Maximum slope values of the split-GFP intensity time trace of *IFIT1-* (black) and *IFIT1+* cells (red) in individual repeats of the experiment shown in Fig. 3c. Dots connected by a line represent values obtained in individual experiments. See method section for explanation about the quantification of the maximal slope. *P* values was determined using two-sided, paired samples T-test. **b**) Maximum slope values of split-GFP time traces from all individual infections in *IFIT1-* and *IFIT1+* cells. Red bars indicate average and s.d. (n = 46 *IFIT1+* and 117 *IFIT1-* infections, 4 independent experiments). *P* value was determined using two-sided, independent samples T-test. **c**) *IFIT1* transcriptional output in infected and uninfected cells observed in the experiments reported in Figs. 3c, 4a,b (n = 163 infected cells and n = 33 uninfected cells, 3 independent experiments). AUC = area under the curve; a.u. = arbitrary units. **d**) Average split-GFP signal accumulation in cells that activate *IFIT1* transcription (dashed lines) and cells that do not activate *IFIT1* transcription (solid lines) infected using either MOI = 1 (black lines) or MOI = 0.2 (blue lines). Line and light shading represent average and s.e.m. of 3 independent experiments (n = 22 and 38 *IFIT1+* and *IFIT1-* cells, respectively, at MOI = 1, n = 20 and 31 *IFIT1+* and *IFIT1-* cells, respectively, at MOI = 0.2). **e**) Cumulative fraction of cells that have activated *IFIT1* transcription at different time points since the start of phase 3. Line indicates average of 3 independent experiments, error bars = s.e.m. (n = 60 and 51 infections at MOI = 1 and MOI = 0.2, respectively). *P* value was determined using two-sided, independent samples T-test at t = 13 h. **f**) Characteristics of split-GFP Low/Medium and GFP High infections. n indicates the total number of infections that were assigned to either group over 3 independent experiments. Fraction of infections indicates the relative proportion of either type of infection. MSE = mean squared error.

For each infected cell, we determined the moment of initial replication using VIRIM and compared split-GFP intensity time traces of cells that activate *IFIT1* transcription with traces of cells that do not initiate *IFIT1* transcription. This analysis revealed that split-GFP signal increased slower in *IFIT1+* cells (Fig. 3c and Extended Data Fig. 4a–c), indicative of slower replication rates. This difference in split-GFP fluorescence accumulation is already apparent at 5 h after initial replication (Fig. 3c), well before *IFIT1* transcription takes place (Fig. 2d). These findings indicate that the lower average viral load in *IFIT1+* cells is not due to virus-induced antiviral gene expression limiting viral replication but, instead, that the rate of viral replication affects the efficiency of antiviral response activation.

To test whether rapid infection progression in a subset of cells causes reduced antiviral response activation, we set out to experimentally increase the rate of viral infection progression. To achieve this, we performed infections using an MOI of 5 instead of 1 (ref. ^32^). As expected, split-GFP accumulation proceeded faster at higher MOI (Fig. 3d) and interestingly, activation of *IFIT1* transcription was less efficient (Fig. 3e), suggesting that higher replication rates indeed result in less efficient antiviral response activation. When using an MOI of 0.2, antiviral response activation was similar to that observed when using an MOI of 1 (Extended Data Fig. 4d,e), consistent with the fact that the majority of cells are infected by a single viral genome under both MOI conditions.

Although viral replication rates, as determined from split-GFP intensity time traces, were predictive of the ability of cells to activate an antiviral response, the predictive power at the single-cell level was modest. Therefore, we performed more in-depth analysis of split-GFP intensity time traces to better predict antiviral response activation for individual cells on the basis of viral replication rates (that is, on split-GFP expression dynamics). We developed an automated analysis pipeline to measure split-GFP intensities and performed unbiased clustering on the resulting intensity time traces. This clustering approach identified a group of infections (~15% of all infections) that is characterized by rapid split-GFP signal accumulation and high split-GFP plateau intensities (Fig. 3f and Extended Data Fig. 4f). In this group of infections, the majority of cells did not activate an antiviral response (Fig. 3g), demonstrating that in cells exhibiting high viral replication rates antiviral response activation is impaired. Together, these findings indicate that heterogeneity in viral replication rates shape the host cell’s ability to activate the antiviral response.

### Efficiency of the antiviral response during infection

**Fig. 4:**
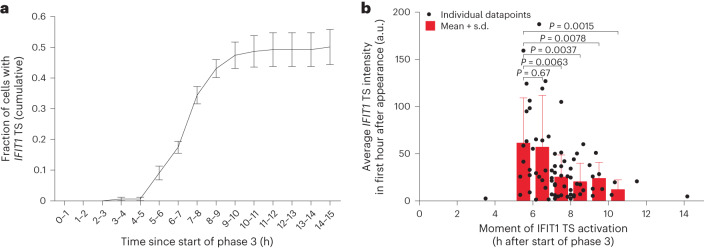
Efficiency of antiviral response activation varies throughout infection. **a**,**b**, For both panels, *24xPBS IFIT1* k.i. cells stably expressing GFP-STAb and PCP-mCherry-NLS were infected with 5xSunTag-EMCV(L^Zn^) and imaged for 16 h. **a**, Cumulative fraction of cells that have activated *IFIT1* transcription at different timepoints since the start of phase 3. Line and error bars indicate mean ± s.e.m. of 4 experiments (*n* = 158 infections). **b**, Scatterplot showing the moment of *IFIT1* transcription activation and average *IFIT1* transcription site intensity in the first hour. Red bars and error bars indicate mean + s.d. *IFIT1* transcription site intensity in different time bins (*n* = 76 cells, 4 independent experiments). *P* values were determined using two-sided, independent-samples *t*-test.

**Extended Data Fig. 5:**
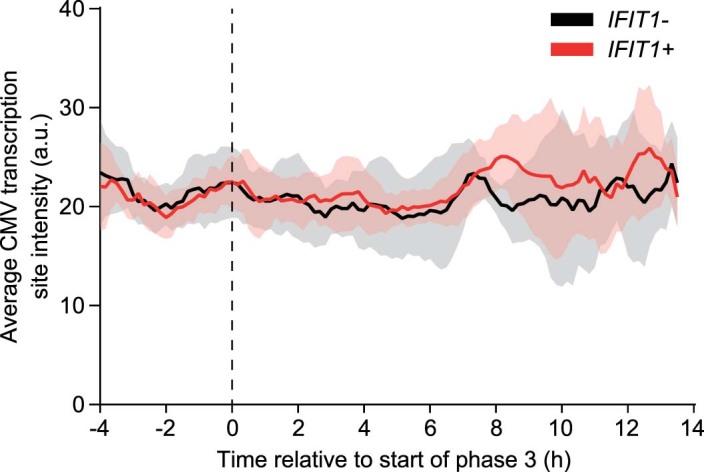
Extended data related to figure 4. *24xPBS IFIT1* cells stably expressing GFP-STAb, PCP-mCherry-NLS, and a CMV-driven 24xPBS reporter RNA were infected with 5xSunTag-EMCV(L^Zn^) and imaged for 16 h. Average CMV transcription site intensities during 5xSunTag-EMCV(L^Zn^) infection in *IFIT1* + (red line) and *IFIT1-* (black line) cells aligned to the start of phase 3 (n = 24 and 24 *IFIT1+* and *IFIT1-* cells, respectively, 3 independent experiments). Shaded areas indicate s.e.m. Source data

The considerable time lag between the first round of viral replication (that is, replication of the incoming vRNA) and the initiation of *IFIT1* transcription (Fig. 2d) suggests that activation of the antiviral response does not occur efficiently early in infection, possibly due to insufficient levels of dsRNA during early infection. To more precisely assess when innate immune activation occurs throughout infection, we determined the onset of *IFIT1* transcription and found that over 90% of the *IFIT1+* cells activate *IFIT1* transcription between 5 and 10 h after initial replication (Fig. 4a). We note that using the *24xPBS IFIT1* reporter, cells that activate *IFIT1* transcription are detected ~90 min earlier compared with experiments using smFISH (Fig. 2d). This difference probably reflects the time required to accumulate 20 mature *IFIT1* transcripts (which we use as a cut-off for *IFIT1* positivity in the smFISH experiments) and demonstrates that the *24xPBS IFIT1* reporter is more sensitive in determining the moment of antiviral gene transcription activation. Interestingly, further examining *IFIT1* transcriptional dynamics, we found that *IFIT1* transcriptional activity was strongest when activated early in infection, with a ~3-fold higher activation level if activated at 5 h vs 10 h after initiation of vRNA replication (Fig. 4b). Importantly, a CMV-driven reporter gene showed constant transcription rates throughout infection (Extended Data Fig. 5), indicating that reduced *IFIT1* transcriptional activation at later timepoints in infection was not due to global virus-induced transcriptional inhibition. Together these findings demonstrate that the efficiency of the antiviral response activation varies during infection.

### Dynamics of IRF3 nuclear translocation during infection

**Fig. 5:**
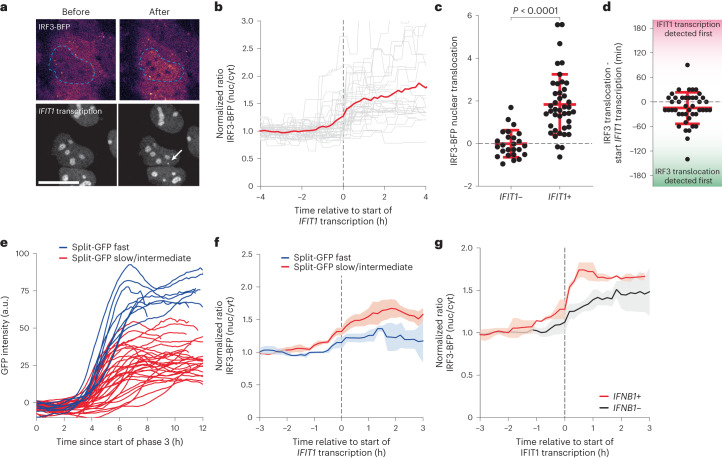
IRF3-BFP nuclear translocation and the antiviral response. **a**–**g**, IRF3-BFP and *24xPBS IFIT1* k.i. cells stably expressing GFP-STAb and PCP-mCherry-NLS were infected with 5xSunTag-EMCV(L^Zn^) and imaged for 16 h. In **e** and **f**, cells additionally expressed GFP1–10 and infection was performed with GFP11-5xSunTag-EMCV(L^Zn^). In **g**, live-cell imaging was followed by smFISH with probes targeting *IFNB1* mRNA. **a**, Representative image of IRF3-BFP localization (top row) before and after *IFIT1* transcription activation (bottom row). Dashed line indicates outline of the nucleus. White arrow in bottom row indicates *IFIT1* transcription site. Scale bar, 20 µm. **b**, Normalized nucleocytoplasmic ratios of IRF3-BFP over time. Time traces of single cells were aligned to the onset of *IFIT1* transcription (*t* = 0) (Methods). Red line indicates the average of all traces and grey lines represent individual traces (*n* = 21 cells, 2 experiments). **c**, IRF3-BFP nuclear translocation efficiency in *IFIT1−* and *IFIT1+* cells. Dots represent individual cells, red lines and error bars indicate mean ± s.d. (*n* = 25 (*IFIT1−*) and 42 (*IFIT1*+) cells, 2 independent experiments). *P* value determined using two-sided, independent-samples *t*-test. **d**, Time between initial *IFIT1* transcription and IRF3-BFP nuclear translocation. Red line and error bars indicate mean ± s.d. (*n* = 44 cells, 3 independent experiments). **e**, Split-GFP intensity time traces of infections synchronized in silico to the start of VIRIM phase 3. Infections were classified as ‘split-GFP fast’ (blue lines) or ‘split-GFP slow/intermediate’ (red lines) by a clustering algorithm. Lines reflect individual cells (*n* = 9 (fast) and 32 (slow/intermediate) infections, 3 independent experiments). **f**, Average normalized nucleocytoplasmic ratios of IRF3-BFP in split-GFP fast (blue line) or slow/intermediate (red line) infections. Traces were synchronized to the start of *IFIT1* transcription. Lines and shaded areas indicate mean ± s.e.m. from 3 experiments (*n* = 9 (fast) and 32 (slow/intermediate) infections). **g**, Normalized nucleocytoplasmic ratios of IRF3-BFP in *IFNB1*+ (red line) and *IFNB1−* (black line) cells aligned to the onset of *IFIT1* transcription. Solid lines and shaded areas indicate mean ± s.e.m. from 3 experiments (*n* = 16 (*IFNB1*+) and 29 (*IFNB1−*) cells, 3 independent experiments).

**Extended Data Fig. 6:**
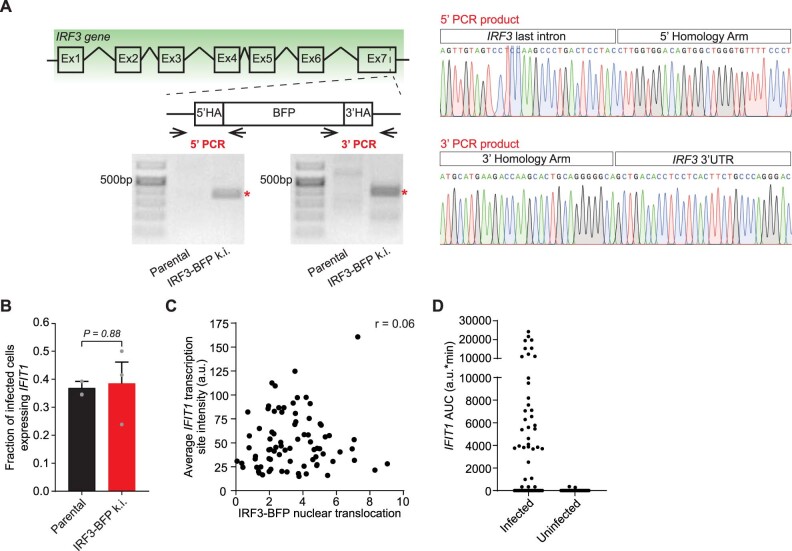
Extended data related to figure 5. **a**) Schematic representation of the *BFP* knock-in in the *IRF3* gene locus and genotyping results. To assess correct integration of the *BFP* coding sequence, a 5’ and 3’ PCR fragment was amplified from genomic DNA using a primer that binds to the *IRF3* genomic sequence and a primer that binds to the *BFP* sequence. PCR reactions were analysed by gel electrophoresis and PCR fragments of correct size (indicated with red asterisk) were subjected to Sanger sequencing (right panel). **b**) Fraction of EMCV(L^Zn^) infected IRF3-BFP or parental HeLa cells expressing >20 *IFIT1* mRNAs at 16 h.p.i. Bars and error bars indicate average ± s.e.m. (n = 426 and 399 infected cells for HeLa and IRF3-BFP, respectively, in 3 independent experiments). *P* value was determined using two-sided, independent samples T-test. Grey dots in B represent values determined in individual biological replicate experiments. **c**) Scatter plot showing the magnitude of IRF3-BFP nuclear translocation and the average *IFIT1* transcription site intensity. IRF3-BFP, *24xPBS IFIT1* cells were infected with GFP11-5xSunTag-EMCV(L^Zn^) at imaged for 16 h. See Methods for detailed explanation on the quantification. r indicates Pearson’s correlation coefficient (n = 80 cells, 6 independent experiments). **d**) *IFIT1* transcriptional output in infected and uninfected cells observed in the experiments reported in Fig. 5a-d, g (n = 57 infected cells and n = 37 uninfected cells in 3 independent experiments).

The absence of antiviral gene expression in a subset of cells may result from inefficient activation of the dsRNA sensing pathway or, alternatively, from inefficient transcriptional activation of antiviral genes. To investigate these possibilities, we set out to monitor antiviral pathway activation by visualizing nuclear translocation of IRF3, a key event in the dsRNA sensing pathway. We tagged the endogenous IRF3 protein with BFP using the CRISPR/Cas9 system (Extended Data Fig. 6a). The resulting IRF3-BFP cell line showed similar levels of innate immune activation as unmodified cells (Extended Data Fig. 6b), indicating that the antiviral response is not affected by this genetic modification. Imaging fluorescent IRF3-BFP translocation in the *24xPBS IFIT1* cell line (Fig. 5a and Supplementary Video 3), revealed that (1) IRF3 nuclear translocation is almost exclusively observed in cells that activate *IFIT1* transcription (Fig. 5b,c) and (2) *IFIT1* transcription is typically activated very shortly after IRF3-BFP nuclear translocation is observed (on average, 15 min after translocation) (Fig. 5d). These findings suggest that the lack of IFIT1 expression in a subset of cells is due to inefficient IRF3 nuclear translocation rather than to inefficient transcriptional activation of the *IFIT1* locus, possibly as a consequence of poor activation of the dsRNA sensing pathway or because of potent antagonism exerted by the virus. Moreover, since IRF3 nuclear translocation temporally coincides with *IFIT1* transcriptional activation (Fig. 5b,d), these results show that the lag between initial viral replication and *IFIT1* expression (Figs. 2d and 4a) is caused by late activation of the viral sensing pathway, rather than by slow transcription activation of IRF3 target genes.

Next, we set out to determine whether viral replication rates affect the level of dsRNA sensing pathway activation in cells that activate an antiviral response. For this, we classified *IFIT1+* infections as replicating either ‘fast’ or ‘slow/intermediate’ on the basis of split-GFP intensity time traces using the clustering algorithm described before and determined the average IRF3-BFP nuclear translocation for both groups (Fig. 5e,f). This revealed that high viral replication rates are associated with less efficient IRF3 translocation, indicative of impaired activation of the dsRNA sensing pathway. Interestingly, the degree of IRF3 nuclear translocation did not correlate strongly with *IFIT1* transcriptional output (Extended Data Fig. 6c), suggesting that a relatively small amount of nuclear IRF3 is sufficient for maximal transcriptional activation of the *IFIT1* gene. In contrast, when comparing *IFNB1* expression levels with IRF3-BFP nuclear translocation, we found that only cells with an extensive IRF3-BFP nuclear translocation showed *IFNB1* expression (Fig. 5g and Extended Data Fig. 6d). Together, these results show that the antiviral signalling that leads to IRF3 nuclear translocation is the rate-limiting step in antiviral gene expression, that *IFIT1* and *IFNB1* are associated with different levels of IRF3 nuclear translocation and, importantly, that differential IRF3 activation in response to infection probably explains the heterogeneity in transcriptional response to viral infection.

## Discussion

Detection of viruses and subsequent initiation of antiviral gene expression in host cells is required for elimination of viral infection. Sporadic activation of antiviral gene expression in infected cells occurs, but the mechanisms that contribute to heterogeneous antiviral responses are poorly understood. Using a combination of VIRIM and real-time analysis of the antiviral response, we showed that cells in which infection progresses slower are more likely to activate an antiviral response.

Previous studies have mostly failed to detect any correlation between viral load and antiviral response activation^21,23,24,35^. This observed lack of correlation might be due to technical limitations of fixed-cell, single-timepoint measurements to assess viral load and antiviral gene expression. Here we imaged early stages of infection, and detected cell-to-cell variation in the start time of infection and the time between infection and completion of the first replication cycle (Extended Data Fig. 2a). After correcting for this variation, our data show that the antiviral response to EMCV is preferentially activated in cells with a lower viral load (Figs. 2e and 3c), an effect that could not be detected without temporal information on early infection (Extended Data Fig. 2f).

Why does the viral replication rate correlate inversely with antiviral response activation? Higher replication rates result in more rapid accumulation of dsRNA in the cell, providing more viral ligands that can trigger immune activation. Counterintuitively, we find that faster replication and higher dsRNA levels are associated with less efficient antiviral response activation (Figs. 2g and 3c). A likely explanation for this paradox is that in cells in which infection proceeds faster, viruses can impair host antiviral response pathways more rapidly before sufficient viral dsRNA is formed for efficient dsRNA sensing. Although the prime host antagonist of EMCV, that is, the L protein, is inactivated in the recombinant EMCV(L^Zn^) virus used in most of our experiments, additional EMCV proteins have been implicated in suppressing the dsRNA sensing pathway^41,43–45^. Importantly, a lower viral load was also observed in *IFIT1+* cells upon infection with EMCV(L^WT^) (Fig. 2f), indicating that heterogeneity in viral replication similarly affects the efficiency of antiviral response activation when the dsRNA sensing pathway is more efficiently inhibited.

Little is known about the factors that determine viral replication rates and how such factors could lead to variation in replication rates among infected cells. We show that infections progress more rapidly when cells are infected by more than one viral particle (Fig. 3d). However, even in cells infected with a single virus, substantial cell-to-cell heterogeneity is observed. Both host-cell intrinsic and virus-intrinsic factors may affect viral replication rates^30,31^. The molecular basis for differences in replication rates is an important topic of future research.

We show that the antiviral response is not efficiently activated until the mid-phase of infection (~5 h after the first round of vRNA replication) (Figs. 2d and 4a). The inability to activate the antiviral response early in infection may be caused by masking of viral dsRNA, for example, through the formation of viral replication organelles, which could prevent dsRNA detection during the first few hours of infection, until the amount of dsRNA exceeds the shielding capacity of these organelles^53,54^. Alternatively, the relatively low amount of dsRNA present in the cell early in infection could be insufficient to trigger activation of the innate immune response, which would suggest that dsRNA sensing mechanisms are relatively insensitive and require large numbers of dsRNA molecules to become activated. Such low sensitivity might be due to inefficient detection of dsRNA molecules by dsRNA sensors (for example, RLRs) or inefficient relay of dsRNA detection signals to downstream activation of the innate immune pathway (that is, IRF3 translocation). A relatively insensitive dsRNA sensor may have evolved to prevent spurious immune activation by endogenous dsRNA ligands in uninfected cells, protecting cells and tissues from an inappropriate inflammatory response. Notably, differences in IRF3 nuclear translocation efficiency were found to correlate with distinct transcriptional responses, with *IFNB1* expression being associated with stronger IRF3 translocation (Fig. 5g). Irrespective of what causes this late antiviral response activation, our data show that once dsRNA sensing induces IRF3 nuclear translocation, antiviral gene expression occurs reliably and fast (Fig. 5b–d), suggesting that the early steps in innate immune pathway activation represent the bottleneck for activation.

Our study has several limitations. First, we used clonal HeLa cell lines to assess how heterogeneity in virus infection affects antiviral response activation. Although we already observe striking heterogeneity in innate immune activation under these conditions, it is probable that under physiological settings, additional sources of variation originating from the host cell (for example, more pronounced variation in antiviral protein expression, exposure to inflammatory cytokines such as IFN) will further impact the outcome of the virus–host interaction. Second, this work focused on EMCV, a (+)ssRNA virus belonging to the picornavirus family. Picornaviruses have a relatively fast infectious cycle and form large amounts of immunostimulatory dsRNA during infection^55^. For other types of viruses, the kinetics of virus–host competition may be very different.

In summary, we show that the dynamics of viral replication and host-cell sensing underpin variation in the antiviral response. We anticipate that application of our strategies to visualize infection progression and antiviral gene transcription in real-time might provide a generalizable approach to studying the relationship between replication and antiviral response activation for other viruses with different replication kinetics, with different evasion mechanisms and in different cell types.

## Methods

### Cell lines

HeLa, HEK293T and BHK-T7 cells were cultured in DMEM (GIBCO) supplemented with 10% FCS (Sigma-Aldrich) and 1% PenStrep (GIBCO). HeLa MDA5 and MAVS k.o. cell lines were previously established^56,57^. Cells were cultured at 37 °C and 5% CO_2_. Cell lines used in this study were routinely tested for presence of mycoplasma.

### Chemicals

The following inhibitors were used in this study: dipyridamole (DiP, 25 μM, Sigma-Aldrich), MRT67307 (MRT, 1 μM, Sigma-Aldrich), Tofacitinib (TOFA, 1 μM, Sigma-Aldrich). All inhibitors were added to cells 30 min before virus addition.

### Virus design and production

5xSunTag-EMCV was produced as described previously^37^. Briefly, a 5xSunTag array was introduced in the infectious pM16.1 complementary (c)DNA clone of the Mengovirus strain of EMCV (kindly provided by A. Palmenberg)^58^. The array was introduced after codon 6 of the Leader protein and was followed by a 3C(D) cleavage sequence (VFETQG) to allow release from the viral polyprotein and prevent the SunTag array from interfering with viral protein functions.

For GFP11-5xSunTag-EMCV, the GFP11 coding sequence was inserted upstream of the 5xSunTag array using Gibson assembly with an annealed oligo pair. No additional cleavage sequence was introduced between the GFP11 and 5xSunTag sequence. To enable infectious RNA production in T7-expressing BHK cells, a T7 terminator sequence was introduced downstream of the viral polyA sequence using Gibson assembly with annealed oligos.

Virus stocks were generated by either transfecting purified in vitro transcribed viral RNA (HiScribe, NEB) or transfecting the infectious cDNA clone containing plasmid in BHK-T7 cells. The day after transfection medium was refreshed and 2–4 d after transfection, when a substantial cytopathic effect was observed, remaining cells and the supernatant were collected and subjected to 3 cycles of freezing–thawing. Cellular debris was cleared by centrifugation and supernatants were collected. Virus titres were determined by endpoint titration and viral RNA was extracted from particles to verify the insert sequence by RT–PCR and Sanger sequencing.

### Cell culture and infection for live imaging

One day before imaging, cells were seeded on a 96-well glass-bottom plate (Matriplates, Brooks) such that cells were at ~80% confluency at the start of imaging. Medium was replaced with Leibovitz’s L15 medium (GIBCO) supplemented with 10% FCS and 1% PenStrep 30 min before the start of imaging. An MOI of ~1 was used for all imaging experiments, except when explicitly stated otherwise.

### Reporter cell line generation

#### (Lentiviral) transduction

GFP-STAb, PCP-mCherry-NLS and GFP(1–10)-P2A-PuroR were introduced into cells using lentiviral infection. For this, pHR-based lentiviral plasmids containing these transgenes were transfected into HEK293T cells together with pMD2G and psPAX2 helper plasmids using Fugene (Promega). After 2 d, viral supernatant was passaged over a 0.45 μm filter to remove cellular debris and polybrene (2 μg ml^−1^, Santa Cruz) was added before transferring the virus-containing supernatant to recipient HeLa cells. At 2 d after virus transfer, medium was replaced and after two passages, single cells from the polyclonal cell population were sorted in 96-well plates by FACS. Cells with GFP-STAb expression were selected to have a similar GFP intensity as a previously established U2OS-GFP-STAb monoclonal cell line that is routinely used for translation imaging in our lab^59^. Cells with PCP-mCherry-NLS expression were sorted for low mCherry fluorescence. GFP(1–10)-P2A-PuroR transduced cells were treated with puromycin (1 μg ml^−1^, Thermo Fisher) 3 d before sorting. After expansion, correct monoclonal cell lines were selected on the basis of expression levels of the transgenes.

To generate a doxycycline-inducible, CMV-promoter-driven, 24xPBS transcription reporter, PCP-mCherry-NLS-expressing HeLa cells were infected with lentiviral particles to express TetR-2A-HygroR. At 2 d after infection, cells were selected for hygromycin resistance using 200 μg ml^−1^ hygromycin (Invivogen). A pcDNA3-based plasmid containing the CMV-TetOn-24xPBS transcription reporter was transfected into the TetR-expressing HeLa cells using Fugene according to manufacturer instructions. At 2 d after transfection, medium was replaced and cells with stable integration of the plasmid were selected using zeocin for 2 weeks (0.4 mg ml^−1^, Invitrogen). Surviving cells were then sorted as single cells in 96-well plates and expanded to generate monoclonal cell lines. Individual clones were screened using doxycycline stimulation (1 μg ml^−1^, Sigma-Aldrich): appropriate clones were selected that had no PP7 transcription site (TS) before doxycycline addition and which presented a transcription site in the nucleus after doxycycline addition.

#### CRISPR/Cas9 genome editing

CRISPR/Cas9 genome editing was used to introduce the 24xPBS transcription reporter and BFP sequence into the *IFIT1* and *IRF3* locus, respectively. In addition to the 24xPBS, for selection purposes, a SNAP-tag and puromycin resistance cassette was included in the knock-in cassette (Extended Data Fig. 3a). The dsDNA donor template for homology-dependent repair was created by excision of the donor sequence from a plasmid using Sap1 restriction enzyme. The ends of the donor sequence contained 300 bp homology to the genomic sequence of the *IFIT1* and *IRF3* loci. The 24 PBS hairpins in the reporter were re-designed to remove stop codons from the coding sequence and a P2A-PuroR-P2A-SNAP-tag-P2A cassette was added to the reporter in frame with the downstream *IFIT1* coding sequence.

Guide RNA (gRNA) sequences were ligated in a Cas9 plasmid (PX459) that was linearized with Bbs1 and from which the puromycin resistance gene was removed. The following guide sequences were used: *IFIT1* 5′-TGATTTAGAAAACAGAGTC-3′, *IRF3* 5′-CATGGATTTCCAGGGCCCTG-3′. The Cas9-guide construct was transfected together with linearized donor template and 2 d after, transfection medium was replaced.

For the *24xPBS IFIT1* k.i., cells were then stimulated with 100 U ml^−1^ recombinant IFNα2 (Sigma-Aldrich) to induce transcription from the *IFIT1* loci, resulting in expression of the puromycin resistance gene, which was integrated into the *IFIT1* locus along with the PBS array. After 24 h, cells were continuously cultured in the presence of puromycin while receiving fresh IFN-containing medium once every 2 d. Surviving cells were expanded and sorted as single cells in 96-well plates to generate monoclonal cell lines. Genomic DNA of expanded clones was extracted using proteinase K digestion and correct integration of the 24xPBS reporter was confirmed by PCR amplification and sequencing of the edited allele. We determined that the second untagged *IFIT1* allele in this cell line is transcribed but does not encode a functional protein because of a frameshifting insertion directly downstream of the translation start site (Extended Data Fig. 3b). For the IRF3-BFP cells, no selection was performed before single-cell sorting by FACS. Monoclones were expanded and screened for BFP expression under the microscope. Correct integration of the *BFP* sequence was confirmed by PCR amplification of the edited locus and sequencing.

#### Validation of 24xPBS IFIT1 knock-in reporter cell line

To validate that expression of the PP7-tagged *IFIT1* allele accurately reports on *IFIT1* expression, we performed dual labelling smFISH with one set of probes targeting the tagged *IFIT1* allele specifically and a second set (complimentary to the *IFIT1* coding sequence) targeting mRNAs originating from both the PP7-tagged and untagged *IFIT1* alleles (Extended Data Fig. 3c). Using this method, we find that the reporter cell line has a single integration site of the 24xPBS reporter sequence because most cells showed only a single transcription site (Extended Data Fig. 3d) (cells in which two 24xPBS reporter transcription sites were detected were likely to be in G2 phase of the cell cycle). Moreover, the transcription site labelled by the reporter-sequence-specific probes co-localized with *IFIT1*-specific probes, indicating that the PBS array had integrated into the *IFIT1* locus (Extended Data Fig. 3c). To determine whether the tagged allele was expressed similarly as the untagged allele, we compared the number of mRNAs expressed from both alleles upon viral infection. We find a strong correlation between the expression levels of untagged *IFIT1* mRNA and PP7-tagged *IFIT1* mRNA (Extended Data Fig. 3e), demonstrating that tagging of the *IFIT1* allele does not alter its expression.

We tested whether live-cell analysis of the PP7-tagged *IFIT1* allele accurately reported on *IFIT1* transcription. For this, we combined live-cell imaging of *IFIT1* transcription with subsequent smFISH of the same cells. We find a strong correlation between cells showing *IFIT1* transcription in live-cell imaging and cells showing *IFIT1* expression by smFISH (Extended Data Fig. 3f). Finally, we tested whether all cells with the *24xPBS IFIT1* k.i. could activate transcription of the reporter allele. Confirming this, we find that upon IFN stimulation (1,000 U ml^−1^), the vast majority of cells (~95%) develop a detectable *IFIT1* transcription site (Extended Data Fig. 3g). Together, these results show that the *24xPBS IFIT1* locus allows sensitive and accurate live-cell measurements of *IFIT1* transcription and can therefore be used as a real-time readout to monitor innate immune activation in single cells.

### smFISH

smFISH was performed according to protocols described previously^60,61^.

#### smFISH probe generation

Stellaris probe designer (https://www.biosearchtech.com/support/tools/design-software/stellaris-probe-designer) was used to design probes targeting *IFIT1*, *IFNB1*, *MAVS*, *TBK1*, *MX2* and *Puro-P2A-SNAP* (part of the 24xPBS transcription reporter) mRNA or EMCV vRNA. All probe sets contained 48 target sequences except for *IFNB1* (31 probes) and *Puro-P2A-SNAP* (36 probes). 20-mer oligonucleotides were ordered from Integrated DNA Technologies and pooled (sequences are listed in Supplementary Data Table 1). All oligonucleotide probes targeting a single RNA were combined and labelled with ddUTP-coupled Atto488, Atto-565 or Atto-633 dyes (AttoTec) using terminal deoxynucleotidyl transferase as described previously^61^. Fluorescent probes were purified by ethanol precipitation, washed to remove unlabelled probes and resuspended in nuclease-free water. Concentration of labelled probes and labelling efficiency were determined by UV/Vis spectroscopy.

#### smFISH staining procedure

Cells on 96-well glass-bottom plates were washed in PBS0 and fixed in 4% formaldehyde (Electron Microscopy Sciences) for 5 min at r.t. After fixation, cells were washed 3 times in PBS0 and permeabilized in 100% ethanol for 30 min on ice, followed by two 15 min washes in wash buffer (2xSSC, 10% formamide in diethyl pyrocarbonate-treated water) at r.t. Labelled smFISH probes were diluted to 10 nM in hybridization buffer (1% dextran sulfate, 2xSSC, 10% formamide in diethyl pyrocarbonate-treated water) and hybridization was performed in a sealed dark container at 37 °C for 16 h. Unbound smFISH probes were removed by two 1 h washes in wash buffer at 37 °C and a 15 min wash at r.t. Samples were stored and imaged in imaging buffer (10 mM Tris pH8, 2xSCC, 0.4% glucose, supplemented with glucose oxidase (Sigma-Aldrich) and catalase (Sigma-Aldrich)). Imaging was performed within 3 d after probe hybridization.

#### smFISH staining in combination with dsRNA immunofluorescence

To combine smFISH staining with immunofluorescence (IF) against dsRNA, the smFISH procedure was followed until the first wash step after probe hybridization. Samples were incubated for 30 min in IF block buffer (PBS0 + 2% BSA (Sigma-Aldrich)) at r.t. Monoclonal anti-dsRNA antibody (J2, Jena Bioscience) diluted at 1:1,000 (from a 1 µg µl^−1^ stock solution) in IF block buffer was added to the samples and incubated for 45 min at r.t. After this, cells were washed 3 times with IF block buffer and incubated in IF block buffer containing 1:500 donkey anti-mouse antibody conjugated to Alexa Fluor 488 (Abcam) for 45 min at r.t. Finally, cells were washed once in IF block buffer, once in smFISH was buffer and samples were stored in smFISH imaging buffer.

### Growth curve and quantitative PCR (RT-qPCR)

HeLa cells expressing GFP-STAb were seeded at 10,000 cells per well in flat-bottom 96-well plates. Cells were infected on the next day for 30 min with 5xST-EMCV(L^Zn^) at MOI = 5. Medium was refreshed at indicated timepoints post infection and plates were either freeze–thawed 3 times to lyse the cells for titration or lysed using RNA lysis buffer for isolation of total RNA for RT-qPCR. Supernatants were titrated with endpoint titration assays to determine viral titres. RT-qPCR was performed to determine the amount of viral RNA copies and expression of *IFIT1* and *IFNB1* at different timepoints post infection. For this, total RNA was isolated using Nucleospin RNA kits (Machery-Nagel). Subsequently, reverse transcription was performed on the isolated RNA using random hexamer primers and *Taq*Man reverse transcriptase (Thermo Fisher). cDNA was subjected to RT-qPCR, with specific primers for *IFIT1*, *IFNB1*, EMCV vRNA and *Actin* (sequences are listed in Supplementary Data Table 1). Relative levels of *IFIT1* and *IFNB1* mRNA and EMCV vRNA were normalized to *Actin* expression. Results are the average of three biological replicates. In each experiment, expression was determined from three technical replicates.

Of note, whereas *IFIT1* induction was first detected at 8–10 h.p.i. by RT-qPCR, it was only detected 7 h after the start of phase 3 by smFISH (Fig. 2d). On average, infections require 6 h to complete phase 2 after addition of virus (Extended Data Fig. 2a) and the induction as observed by smFISH would thus appear to occur considerably later than as detected by RT-qPCR. However, a subset of infections rapidly completes the initial replication (<2.5 h) and the moment of antiviral gene induction as detected by RT-qPCR probably reflects activation of IFIT1 expression in a fraction of these cells.

### Microscopy

All fluorescence microscopy was performed on a Nikon TI2 inverted microscope equipped with a Yokagawa CSU-X1 spinning disc and a Prime 95B sCMOS camera (Photometrics). Imaging was performed using a ×60/1.40 NA oil objective. Image acquisition was performed using NIS Elements software and making use of the ‘perfect focus system’ to correct for *Z* drift during time-lapse imaging experiments. The microscope was equipped with a temperature-controlled incubator and imaging was performed at 37 °C for live-cell experiments or at r.t. for smFISH samples.

#### Microscopy acquisition settings

For smFISH samples that were not previously subjected to live-cell imaging, a random position in the centre of the well was selected and a large field of view was constructed by imaging 4 × 4 neighbouring imaging fields. Approximately 15 *Z*-slices at 0.5 μm interval that covered the entire cell were acquired for *IFIT1*, *IFNB1*, *MAVS*, *TBK1*, *MX2* and *Puro-P2A-SNAP* smFISH labelled cells and a single *Z*-slice at the centre of the cell was acquired for EMCV FISH labelling. smFISH samples were imaged with a 50 ms exposure time (except EMCV FISH for which 70 ms exposure was used).

For live-cell imaging experiments that were not followed by smFISH, random non-overlapping positions were selected. Movies with live-cell reporters were acquired with a 5 min time interval between frames, except for the BFP channel in experiments involving the IRF3-BFP cell line; because of the high laser power required to obtain sufficient BFP signal, we limited the imaging interval to 1 frame per 30 or 60 min to reduce phototoxicity (initial experiments were performed at 1 frame per 60 min to minimize phototoxicity; in later experiments, 1 frame per 30 min was found to be equally well-tolerated by cells). Typically, 10 *Z*-slices at 0.8 μm interval were acquired for GFP and mCherry channels, whereas a single *Z*-slice was acquired for the BFP channel in experiments involving the IRF3-BFP cell line. Signal in the red channel (561 nm laser), used for imaging of *IFIT1* transcription, was acquired using a 50 ms exposure time; signal in the green channel (488 nm laser), used for VIRIM and split-GFP imaging, was acquired with an exposure time of 70 ms; signal in the blue channel (405 nm laser), used to visualize IRF3-BFP localization, was acquired using a 50 ms exposure time.

For live-cell imaging experiments that were followed by smFISH, positions were selected for live imaging in a pattern that could be retrieved. For this, a series of consecutive field-of-views was selected starting from the edge of the imaging well. Images were taken at a 10 min interval. After completion of the live-imaging experiment, the imaging plate was gently removed from the microscope stage and cells were immediately washed and fixed. After completion of smFISH (and IF) staining protocol, positions were retrieved by navigating to the first field of view at the edge of the imaging well and imaged again to visualize smFISH labelling. For imaging of dsRNA IF staining, a stack of fifteen 0.5 µm slices was acquired using a 70 ms exposure time.

#### Post-acquisition data processing

Maximal intensity projections for all *Z*-slices were generated using NIS Elements software and all downstream analyses were performed on these projections. In experiments involving intensity measurements (split-GFP signal accumulation, *IFIT1* transcription and IRF3-BFP nuclear translocation), analysed channels were corrected for photobleaching using the ‘bleach correction’ plugin in ImageJ.

### Data analysis

#### smFISH analysis

To calculate the fraction of infected cells that was positive for antiviral gene expression, the number of infected cells was first determined on the basis of the EMCV FISH signal. However, only if a cell had >50 EMCV smFISH spots was it considered infected because at 16 h.p.i., considerable release of viral particles from infected cells resulted in substantial smFISH signal originating from virus particles on the outside of a cell. Beyond ~8 h.p.i., the amount of viral genomes in infected cells was frequently too high to count individual vRNAs. These cells were also scored as EMCV positive. In the experiment with dipyridamole treatment (Fig. 1e), no cells with >50 vRNAs could be detected and instead, all cells in the large image were evaluated for *IFIT1* and *IFNB1* expression (again, viral particles on the outside of the cell precluded accurate quantification of the number of vRNAs in the cell by smFISH). The number of *IFIT1* and/or *IFNB1* mRNA spots was determined for each infected cell. To determine the number of nascent RNAs (Extended Data Fig. 1f), only the spots overlapping with the nuclear marker BFP-NLS were determined. The number of spots was determined using the ‘Spot Counter’ plugin in ImageJ. Detection settings were optimized for each measurement, due to experimental variation in smFISH labelling, and manually curated for each measurement. For *IFIT1*, *IFNB1*, *MAVS*, *TBK1*, *MX2* and *Puro-P2A-SNAP* smFISH, spots that were unusually bright (>2.5-fold mean intensity of single spots) and non-spherical were not scored as individual mRNAs, as such foci probably originated from dye aggregates. Transcription sites identification based on smFISH signal (Extended Data Fig. 3d) was defined as any spherical foci, localized in the nucleus, with a high spot intensity (>2.5-old mean intensity of single spots). At ~200 spots per cell, considerable overlap in spots in the maximum intensity projection impaired accurate spot detection, hence 200 spots per cell was set as an upper limit for quantification. On the basis of the number of smFISH spots in uninfected cells, we set a stringent cut-off value of 20 *IFIT1*, 10 *IFNB1* and 5 *MX2* mRNAs, above which a cell was considered positive for expression of each gene.

If cells were partially outside the field of view or if cells were blurred at an image stitch during large-image construction, they were not included in the analysis.

#### Viral load and dsRNA IF intensity measurement

Viral load was determined using the cytosolic fluorescence intensity of EMCV-Atto488 FISH staining. The mean intensities of 10–20 regions of interest (ROIs) (20 × 20 pixels) that were randomly positioned in the cytoplasm without overlap were determined and the average of the measurements was calculated. The number of ROI measurements was chosen so that >80% of the cell’s cytoplasm was ultimately part of an intensity measurement. For each repeat, an average cellular background signal intensity was derived from uninfected cells and subtracted from average cellular EMCV signal intensities. To compare viral loads between different experiments, these values were normalized to the average intensity of the 10 cells with the highest signal in the experiment and this normalized value was multiplied by 1,000.

Note that Fig. 2e first shows a notable increase in viral load only at 4 h, even though the smFISH-based approach has single-genome detection sensitivity. This apparent absence of signal is due to our quantification method: because individual vRNAs cannot be resolved at late infection timepoints, we quantified viral load using total FISH staining intensity rather than smFISH spot count. Since the fluorescence signal of a single vRNA contributed very little to total cellular fluorescence, an increase in viral load was only observed when large numbers of vRNAs were present in the cell.

To quantify dsRNA IF staining intensity, average cellular signal intensity was determined. The average signal derived from uninfected cells was subtracted from each intensity, yielding a background-subtracted intensity. These values were normalized per experiment similar to how viral load was normalized (that is, to the average of the 10 cells with the highest signal).

#### smFISH spot intensity

To determine the distribution of *IFIT1* and *IFNB1* smFISH spot intensities, the intensity of all spots (max. 30 per cell) in a random region of a *IFIT1/IFNB1+* cell was determined. For this, a 4 × 4 pixel ROI centred around the middle of a spot was used to measure the mean fluorescence intensity. Mean intensity of an adjacent 4 × 4 pixel ROI without spot was determined and subtracted from the spot intensity. Non-spherical/overlapping spots and putative transcription sites were excluded from the analysis. Background-subtracted spot intensities were normalized to the average spot intensity in the cell.

#### VIRIM quantification

Annotation of viral infection phases based on SunTag spots in VIRIM was performed as described previously^37^. In brief, GFP spots were considered viral translation sites on the basis of their size, mobility and intensity. For example, when cells expressed both GFP-STAb and GFP(1–10), a fraction of cells presented with large cytosolic GFP spots that are not viral translation sites (they are present in uninfected cells as well). However, these spots can be readily discriminated from viral translation sites on the basis of their larger size and slower mobility.

The start of VIRIM phase 3 was defined as the moment when one or more viral translation site(s), as visualized by SunTag labelling, re-appear after being absent during VIRIM phase 2 (initial replication phase). Because of the imaging interval of 5 or 10 min and the relatively short duration of phase 3 (~30 min), a steep increase in the number of SunTag spots between frames was typically observed during VIRIM phase 3. This steep increase in the number of SunTag spots was used to pinpoint the start of phase 3 in those cells in which accurate calling of VIRIM phase 1 (and 2) was challenging. In experiments involving infections at MOI = 5, multiple translating incoming vRNAs precluded accurate assignment of VIRIM phases 1 and 2. Nevertheless, the typical ‘bursty’ increase of multiple newly translating vRNAs that mark the start of VIRIM phase 3 can still be observed. Therefore, in MOI = 5 experiments, the start of phase 3 was determined by the first timepoint of a timepoints series during which a steep increase in the number of translating vRNAs was observed.

The start of VIRIM phase 1 was defined as the first timepoint of a series of at least 4 timepoints in which a single translation site was visible in 3 or more timepoints. In Fig. 2h and Extended Data Fig. 2g, infections were classified as ‘abortive infection’ if (1) the start of VIRIM phase 1 occurred in the first 8 h after addition of virus and (2) no VIRIM phase 3 was observed in the remainder of the movie, that is, in the remaining ~8 h.

#### VIRIM in combination with smFISH

Various parameters were determined from the VIRIM live imaging and smFISH staining. Start of VIRIM phase 3, number of IFIT1 and IFNB1 spots and EMCV viral load were determined as described above.

Only cells for which complete VIRIM history and successful smFISH staining were available were analysed. Cells for which time-lapse imaging data could not be faithfully linked to smFISH data (for instance, because of high cell density) were excluded from analysis. Cells that underwent mitosis were excluded from the analysis if mitosis took place just before the start of VIRIM phase 3 (<30 min after completion of cytokinesis) or if mitosis occurred between the start of phase 3 and the end of the movie. For EMCV(L^WT^)-infected cells, only positions in which an IFIT1+ cell was present were analysed.

#### Transcription site intensity measurements

Identification of 24xPBS *IFIT1* transcription sites during live imaging was based on the following criteria: (1) An *IFIT1* TS is a spherical (diffraction limited) spot and considerably smaller in size compared with typical nucleoli (which are also enriched in mCherry signal; see for instance, Fig. 3a). An *IFIT1* TS fits within a 5 × 5 pixel ROI. (2) *IFIT1* TSs show slow and highly confined diffusion within the nucleus. (3) An *IFIT1* TS emerges during the course of infection, that is, they are absent at the start of infection and not present in uninfected cells. (4) The mCherry fluorescence intensity of an *IFIT1* TS fluctuates over time, in contrast to mCherry signal originating from nucleoli and aggregates. (5) An *IFIT* TS is present for a prolonged period (>60 min, in a minimum of 6 frames). The timepoint of the first appearance of an mCherry spot that meets these criteria was considered as the onset of *IFIT1* transcription. Multinucleated cells or cells that formed syncytia during the movie were excluded from analysis.

The intensity of *IFIT1* TSs was determined by measuring the mean intensity of a 5 × 5 pixel ROI positioned over the centre of the TS and subtracting the mean intensity of an ROI of the same size positioned directly adjacent to the TS from this value. If a TS overlaps (partially) with a nucleolus, then background subtraction was performed by measuring the intensity of an ROI in the direct vicinity of the TS that has a comparable fraction of nucleolar overlap. From the intensity time trace, the area under the curve (AUC) was determined using the trapezoidal rule, where the average intensity value between consecutive timepoints was determined and multiplied by the time interval. These values were summed to determine the AUC of an *IFIT1* TS intensity time trace. Cells with AUC > 5,000 a.u. were considered positive for *IFIT1* transcription. To quantify *IFIT1* transcriptional activity at the onset of transcription, the average *IFIT1* TS intensity in the first hour after the appearance of an *IFIT1* TS was determined. If a TS was temporarily absent during this 1 h time period, an intensity value of zero was included in the average calculation.

Intensity of the *24xPBS CMV* TS was determined in a similar fashion to *IFIT1* TS intensity measurements. Compared to *IFIT1*, *CMV* TS identification differed in one aspect: *CMV* TSs are present at the start of the movie and remain present throughout the movie. To be included in the analysis, at least 66% of the frames must have a detectable *CMV* TS. Average *CMV* intensity traces were smoothened by applying a moving average with a window size of 5 timepoints.

#### Split-GFP intensity measurements

While both the VIRIM and split-GFP systems have their readouts in the GFP channel, both can be accurately assessed simultaneously in the same cell because early in infection, the signal originating from the split-GFP is low, allowing readout of the VIRIM signal (that is, GFP foci), while later in infection (~3 h after the start of phase 3), the signal originating from the split-GFP system becomes strong enough to detect over the ‘background’ GFP signal originating from GFP-STAb (Fig. 3b,c).

To measure split-GFP reconstitution, the mean cytosolic GFP intensity was measured at every timepoint. For this, a 25 × 25 pixel ROI was positioned in the perinuclear region of the cell at 3 non-overlapping positions and the average was calculated. If fluorescent aggregates were present in the cell (see also ‘VIRIM quantification’), these were avoided. To subtract baseline GFP signal originating from the GFP-STAb, the average cytosolic GFP intensity in the first 2 h of the movie was subtracted from all values. In some cases, morphological changes to the cell occurred during the movie that strongly affected GFP intensity measurements, for example, during cell death or detachment at the end of infection. In such cases, measurements after the morphological changes occurred were excluded from further analysis.

#### Automated split-GFP measurement and cluster analysis

Nuclear segmentation was performed on the basis of the nuclear signal of PCP-mCherry-NLS using cellpose^62^. The mean GFP pixel intensity for each nuclear mask at each timepoint was computed. Single cells were tracked over time using the btrack algorithm^63^. Segmentation and tracking results were displayed in napari and the performance of the algorithms was manually curated for a subset of positions. A track length of at least 40 timepoints was chosen as a quality threshold, resulting in a total number of 1,430 tracks from 3 independent experiments.

Single-cell split-GFP tracks were smoothed by applying a moving average with a window size of 10 timepoints. The smoothed single-cell traces were then clustered using the dynamic time warping algorithm from the dtw R package^64^. The resulting distance matrix was used for hierarchical clustering using average linkage and split into eight clusters. This resulted in three main clusters corresponding to the non-infected traces (flat-shaped curve, *n* = 440) and two sigmoidal curve shapes, differing in their growth rate and plateau height (split-GFP low/medium *n* = 842, split-GFP high *n* = 129).

Importantly, the dynamic time warping algorithm clusters split-GFP intensity time traces on the basis of trace similarity independent of when infection is initiated. This allows inclusion of infections that started at different moments during the live-cell imaging.

#### IRF3-BFP nuclear translocation

To determine the intensity ratio of nuclear/cytosolic IRF3-BFP, BFP signal intensity was measured in the nucleus and cytosol in the same manner as cytosolic split-GFP signal intensity (that is, the average of 3 mean intensity measurements using a 25 × 25 pixel ROI was determined; see section ‘Split-GFP intensity measurements’). Background correction was performed by subtracting the mean intensity of a 25 × 25 pixel ROI positioned at a cell-free area. The nucleocytosolic IRF3-BFP ratio was determined for every timepoint and time traces were aligned to the start of IFIT1 transcription (or start of phase 3 in the case of Fig. 5c). Ratio time traces were normalized to the average nucleocytosolic ratio between 2 to 7 h before the start of *IFIT1* transcription (or start of phase 3).

To quantify the translocation efficiency, the AUC of the nucleocytosolic ratio time traces before normalization was determined using the trapezoidal rule starting from 6 h after the start of phase 3 until the end of the movie (this timepoint was chosen because at 6 h after the start of phase 3, the first infected cells displayed IRF3-BFP nuclear translocation). The AUC was divided by the number of frames that were included in the AUC calculation to correct for different trace durations.

IRF3-BFP nuclear translocation was defined using the following requirements: (1) An increase in the nucleocytosolic ratio was observed in multiple, consecutive frames (spanning >30 min, in a minimum of 3 frames). (2) During at least two frames, the increase in the nucleocytosolic ratio was at least 0.1 unit. The moment of translocation was defined as the first timepoint of a series of frames that fulfilled these requirements. If no IRF3-BFP nuclear translocation was observed according to these criteria, cells were not included in the analysis to determine the timing difference between the moment of translocation and the start of *IFIT1* transcription (Fig. 5d).

#### Statistical analysis

Unless stated otherwise, statistical tests were performed using a *P* value of 0.05 as a cut-off for significance and assuming normal distribution of experimentally determined averages. Normality was not assumed when comparing endogenous expression levels of *MAVS*, *TBK1* and IRF3 in *IFIT1−* and *IFIT1+* cells (Extended Data Fig. 1g–i) and when comparing the maximum slopes of split-GFP intensity time traces (Extended Data Fig. 4b). In these instances, Mann–Whitney tests were performed to assess statistical significance of distribution differences. All *P* values were calculated using two-tailed tests. The type of test and the type of error bars used in figures are indicated in the figure legends. An overview of the number of experimental repeats and the total number of observations per condition are listed in Supplementary Data Table 2. Genotyping results presented in Extended Data Fig. 3b show representative results of two repeat experiments.

To extract descriptive parameters from the split-GFP intensity time traces (Extended Data Fig. 4a,b,f), a logistic growth curve was fitted on the (average) split-GFP traces using the following general equation:

1 $$f\left(x\right)=\frac{A}{1+B {e}^{\left(-C t\right)}}$$

where *f (x)* describes the split-GFP intensity as a function of time (*t*), A represents the plateau value, *B* is a baseline-derived constant and *C* is the logistic growth rate. The maximum slope was calculated from the plateau value and *C* parameter using the following equation:

2 $${\rm{Max}.{slope}}=A \frac{C}{4}$$

The mean squared error (MSE) was calculated to determine the quality of fit.

(Linear) regression analysis was performed in GraphPad PRISM. To quantify the extent of correlation in Fig. 1g and Extended Data Figs. 1f, 2f and 5c, the Pearson’s correlation coefficient (*r*) was determined. In Extended Data Fig. 3e, linear regression was performed excluding observations where the number of mRNAs was above the detection limit (>200 mRNAs). The 95% confidence interval of the linear fit and the coefficient of determination (*R*^2^) were determined to assess the quality of the linear regression.

### Reporting summary

Further information on research design is available in the Nature Portfolio Reporting Summary linked to this article.

## Supplementary information

Reporting Summary

Supplementary Video 1 Movie of live-cell virus imaging using VIRIM combined with smFISH for *IFIT1*, *IFNB1* and EMCV. HeLa cells expressing GFP-STAb were infected with 5xSunTag-EMCV(L^Zn^) and imaged for 16 h. Then, cells were fixed and smFISH staining was performed for *IFNB1* mRNA (magenta), *IFIT1* mRNA (cyan) and positive strand EMCV vRNA (EMCV(+), white). For the VIRIM imaging, a maximal intensity projection of 9 *Z*-slices was generated. Time is indicated in h:min since the start of image acquisition. Images of the smFISH staining were projected on top of the last frame of the VIRIM imaging.

Supplementary Video 2 Movie of live-cell virus imaging using VIRIM and split-GFP in combination with *IFIT1* transcription imaging. *24xPBS IFIT1* cells expressing GFP-STAb, GFP(1–10) and PCP-mCherry-NLS were infected with GFP11-5xSunTag-EMCV(L^Zn^) and imaged for 16 h. Left panel (green) shows VIRIM and split-GFP signal, middle panel (magenta) shows *IFIT1* transcription signal (first appearance of *IFIT1* TS at 11h10) and the right panel shows the merge of both imaging channels. For both channels, 9 *Z*-slices were acquired and processed to generate a maximal intensity projection. In the GFP channel, large immobile foci can be observed which probably represent aggregates of GFP-STAb and GFP1–10 (see Methods). Time is indicated in h:min since the start of image acquisition. Images from this movie are also shown in the example images in Fig. 3a.

Supplementary Video 3 Movie of live-cell imaging of *IFIT1* transcription and IRF3-BFP nuclear translocation. IRF3-BFP and *24xPBS IFIT1* cells stably expressing GFP-STAb and PCP-mCherry-NLS were infected with 5xSunTag-EMCV(L^Zn^) and imaged for 16 h. Left panel (in grey) shows *IFIT1* transcription signal (first appearance of *IFIT1* TS at 13h00) and the right panel (displayed using the ‘inferno’ lookup table) shows the IRF3-BFP signal. For the *IFIT1* transcription imaging, 9 *Z*-slices were acquired every 10 min and processed to generate a maximal intensity projection. For IRF3-BFP, a single *Z*-slice was acquired every 60 min. Time is indicated in h:min since the start of image acquisition. Images from this movie are also shown in the example images in Fig. 5a.

Supplementary Data Tables 1 and 2 Table 1. List of oligonucleotides. Table 2. Number of repeats and observations.

## Source data

Source Data Extended Data Fig. 3 Unprocessed gel images.

Source Data Extended Data Fig. 5 Unprocessed gel images.

## Data Availability

A selection of source imaging data for all figures is publicly available at Mendeley data: 10.17632/8p8vy5s35b.1. Source data are provided with this paper.

## Code availability

The Python code used for automated quantification and clustering of split-GFP intensity time traces used in Figs. 3f and 5f, and the code used for performing the logistic fits in Extended Data Fig. 4a,b,f are available at Mendeley data: 10.17632/8p8vy5s35b.1.

## References

[1] O’Neill, L. A. J. & Bowie, A. G. Sensing and signaling in antiviral innate immunity. *Curr. Biol.* **20**, R328–R333 (2010). 20392426 10.1016/j.cub.2010.01.044

[2] Schoggins, J. W. & Rice, C. M. Interferon-stimulated genes and their antiviral effector functions. *Curr. Opin. Virol.* **1**, 519–525 (2011). 22328912 10.1016/j.coviro.2011.10.008 PMC3274382

[3] Mesev, E. V., LeDesma, R. A. & Ploss, A. Decoding type I and III interferon signalling during viral infection. *Nat. Microbiol.* **4**, 914–924 (2019). 30936491 10.1038/s41564-019-0421-x PMC6554024

[4] Postal, M. et al. Type I interferon in the pathogenesis of systemic lupus erythematosus. *Curr. Opin. Immunol.* **67**, 87–94 (2020). 33246136 10.1016/j.coi.2020.10.014 PMC8054829

[5] Lee, J. S. & Shin, E.-C. The type I interferon response in COVID-19: implications for treatment. *Nat. Rev. Immunol.* **20**, 585–586 (2020). 32788708 10.1038/s41577-020-00429-3 PMC8824445

[6] Sposito, B. et al. The interferon landscape along the respiratory tract impacts the severity of COVID-19. *Cell* **184**, 4953–4968.e16 (2021). 34492226 10.1016/j.cell.2021.08.016 PMC8373821

[7] Nelemans, T. & Kikkert, M. Viral innate immune evasion and the pathogenesis of emerging RNA virus infections. *Viruses* **11**, 961 (2019). 31635238 10.3390/v11100961 PMC6832425

[8] Crow, Y. J. Type I interferonopathies: a novel set of inborn errors of immunity. *Ann. N. Y. Acad. Sci.* **1238**, 91–98 (2011). 22129056 10.1111/j.1749-6632.2011.06220.x

[9] Crow, Y. J. & Stetson, D. B. The type I interferonopathies: 10 years on. *Nat. Rev. Immunol.*10.1038/s41577-021-00633-9 (2021). 34671122 10.1038/s41577-021-00633-9 PMC8527296

[10] Pichlmair, A. et al. Activation of MDA5 requires higher-order RNA structures generated during virus infection. *J. Virol.* **83**, 10761–10769 (2009). 19656871 10.1128/JVI.00770-09 PMC2753146

[11] Dias Junior, A. G., Sampaio, N. G. & Rehwinkel, J. A. Balancing act: MDA5 in antiviral immunity and autoinflammation. *Trends Microbiol.* **27**, 75–85 (2019). 30201512 10.1016/j.tim.2018.08.007 PMC6319154

[12] Rehwinkel, J. & Gack, M. U. RIG-I-like receptors: their regulation and roles in RNA sensing. *Nat. Rev. Immunol.* **20**, 537–551 (2020). 32203325 10.1038/s41577-020-0288-3 PMC7094958

[13] Andersen, J., VanScoy, S., Cheng, T.-F., Gomez, D. & Reich, N. C. IRF-3-dependent and augmented target genes during viral infection. *Genes Immun.* **9**, 168–175 (2008). 18094709 10.1038/sj.gene.6364449

[14] Savitsky, D., Tamura, T., Yanai, H. & Taniguchi, T. Regulation of immunity and oncogenesis by the IRF transcription factor family. *Cancer Immunol. Immunother.* **59**, 489–510 (2010). 20049431 10.1007/s00262-009-0804-6 PMC11030943

[15] Schoggins, J. W. Interferon-stimulated genes: what do they all do? *Annu. Rev. Virol.* **6**, 567–584 (2019). 31283436 10.1146/annurev-virology-092818-015756

[16] Schneider, W. M., Chevillotte, M. D. & Rice, C. M. Interferon-stimulated genes: a complex web of host defenses. *Annu. Rev. Immunol.* **32**, 513–545 (2014). 24555472 10.1146/annurev-immunol-032713-120231 PMC4313732

[17] Grandvaux, N. et al. Transcriptional profiling of interferon regulatory factor 3 target genes: direct involvement in the regulation of interferon-stimulated genes. *J. Virol.* **76**, 5532–5539 (2002). 11991981 10.1128/JVI.76.11.5532-5539.2002 PMC137057

[18] Lei, X., Xiao, X. & Wang, J. Innate immunity evasion by enteroviruses: insights into virus–host interaction. *Viruses* **8**, 22 (2016). 26784219 10.3390/v8010022 PMC4728582

[19] Feng, Q., Langereis, M. A. & van Kuppeveld, F. J. M. Induction and suppression of innate antiviral responses by picornaviruses. *Cytokine Growth Factor Rev.* **25**, 577–585 (2014). 25086453 10.1016/j.cytogfr.2014.07.003 PMC7172595

[20] Rand, U. et al. Uncoupling of the dynamics of host–pathogen interaction uncovers new mechanisms of viral interferon antagonism at the single-cell level. *Nucleic Acids Res.* **42**, e109 (2014). 24895433 10.1093/nar/gku492 PMC4117750

[21] Doğanay, S. et al. Single-cell analysis of early antiviral gene expression reveals a determinant of stochastic IFNB1 expression. *Integr. Biol.* **9**, 857–867 (2017). 10.1039/c7ib00146k PMC6201300 29098213

[22] Zhao, M., Zhang, J., Phatnani, H., Scheu, S. & Maniatis, T. Stochastic expression of the interferon-β gene. *PLoS Biol.* **10**, e1001249 (2012). 22291574 10.1371/journal.pbio.1001249 PMC3265471

[23] Drayman, N., Patel, P., Vistain, L. & Tay, S. HSV-1 single-cell analysis reveals the activation of anti-viral and developmental programs in distinct sub-populations. *eLife* **8**, e46339 (2019). 31090537 10.7554/eLife.46339 PMC6570482

[24] Patil, S. et al. Single-cell analysis shows that paracrine signaling by first responder cells shapes the interferon-β response to viral infection. *Sci. Signal.* **8**, ra16 (2015). 25670204 10.1126/scisignal.2005728

[25] Zawatzky, R., De Maeyer, E. & De Maeyer-Guignard, J. Identification of individual interferon-producing cells by in situ hybridization. *Proc. Natl Acad. Sci. USA* **82**, 1136–1140 (1985). 3856251 10.1073/pnas.82.4.1136 PMC397209

[26] Sjaastad, L. E. et al. Distinct antiviral signatures revealed by the magnitude and round of influenza virus replication in vivo. *Proc. Natl Acad. Sci. USA* **115**, 9610–9615 (2018). 30181264 10.1073/pnas.1807516115 PMC6156629

[27] Wimmers, F. et al. Single-cell analysis reveals that stochasticity and paracrine signaling control interferon-alpha production by plasmacytoid dendritic cells. *Nat. Commun.* **9**, 3317 (2018). 30127440 10.1038/s41467-018-05784-3 PMC6102223

[28] Rand, U. et al. Multi-layered stochasticity and paracrine signal propagation shape the type-I interferon response. *Mol. Syst. Biol.* **8**, 584 (2012). 22617958 10.1038/msb.2012.17 PMC3377992

[29] Talemi, S. R. & Höfer, T. Antiviral interferon response at single-cell resolution. *Immunol. Rev.* **285**, 72–80 (2018). 30129203 10.1111/imr.12699

[30] Jones, J. E., Le Sage, V. & Lakdawala, S. S. Viral and host heterogeneity and their effects on the viral life cycle. *Nat. Rev. Microbiol.* **19**, 272–282 (2021). 33024309 10.1038/s41579-020-00449-9 PMC7537587

[31] Guo, F. et al. Single-cell virology: on-chip investigation of viral infection dynamics. *Cell Rep.* **21**, 1692–1704 (2017). 29117571 10.1016/j.celrep.2017.10.051 PMC5689460

[32] Schulte, M. B. & Andino, R. Single-cell analysis uncovers extensive biological noise in poliovirus replication. *J. Virol.* **88**, 6205–6212 (2014). 24648454 10.1128/JVI.03539-13 PMC4093869

[33] Russell, A. B., Trapnell, C. & Bloom, J. D. Extreme heterogeneity of influenza virus infection in single cells. *eLife* **7**, e32303 (2018). 29451492 10.7554/eLife.32303 PMC5826275

[34] Fiege, J. K. et al. Single cell resolution of SARS-CoV-2 tropism, antiviral responses, and susceptibility to therapies in primary human airway epithelium. *PLoS Pathog.* **17**, e1009292 (2021). 33507952 10.1371/journal.ppat.1009292 PMC7872261

[35] O’Neal, J. T. et al. West Nile virus-inclusive single-cell RNA sequencing reveals heterogeneity in the Type I interferon response within single cells. *J. Virol.* **93**, e01778-18 (2019). 30626670 10.1128/JVI.01778-18 PMC6401468

[36] Martin, B. E., Harris, J. D., Sun, J., Koelle, K. & Brooke, C. B. Cellular co-infection can modulate the efficiency of influenza A virus production and shape the interferon response. *PLoS Pathog.* **16**, e1008974 (2020). 33064776 10.1371/journal.ppat.1008974 PMC7592918

[37] Boersma, S. et al. Translation and replication dynamics of single RNA viruses. *Cell* **183**, 1930–1945.e23 (2020). 33188777 10.1016/j.cell.2020.10.019 PMC7664544

[38] Satoh, T. et al. LGP2 is a positive regulator of RIG-I- and MDA5-mediated antiviral responses. *Proc. Natl Acad. Sci. USA* **107**, 1512–1517 (2010). 20080593 10.1073/pnas.0912986107 PMC2824407

[39] Deddouche, S. et al. Identification of an LGP2-associated MDA5 agonist in picornavirus-infected cells. *eLife* **3**, e01535 (2014). 24550253 10.7554/eLife.01535 PMC3967861

[40] Fout, G. S. & Simon, E. H. Antiviral activities directed against wild-type and interferon-sensitive mengovirus. *J. Gen. Virol.* **64**, 1543–1555 (1983). 6190989 10.1099/0022-1317-64-7-1543

[41] Hato, S. V. et al. The mengovirus leader protein blocks interferon-alpha/beta gene transcription and inhibits activation of interferon regulatory factor 3. *Cell Microbiol.* **9**, 2921–2930 (2007). 17991048 10.1111/j.1462-5822.2007.01006.x

[42] Tanenbaum, M. E., Gilbert, L. A., Qi, L. S., Weissman, J. S. & Vale, R. D. A protein-tagging system for signal amplification in gene expression and fluorescence imaging. *Cell* **159**, 635–646 (2014). 25307933 10.1016/j.cell.2014.09.039 PMC4252608

[43] Huang, L. et al. Encephalomyocarditis virus 3C protease attenuates type I interferon production through disrupting the TANK-TBK1-IKKε-IRF3 complex. *Biochem. J.* **474**, 2051–2065 (2017). 28487378 10.1042/BCJ20161037 PMC5465970

[44] Li, L. et al. Encephalomyocarditis virus 2C protein antagonizes interferon-β signaling pathway through interaction with MDA5. *Antivir. Res.* **161**, 70–84 (2019). 30312637 10.1016/j.antiviral.2018.10.010

[45] Han, Y. et al. Encephalomyocarditis virus abrogates the interferon beta signaling pathway via its structural protein VP2. *J. Virol.* **95**, e01590-20 (2021). 33328314 10.1128/JVI.01590-20 PMC8094936

[46] Bandyopadhyay, S. K., Leonard, G. T., Bandyopadhyay, T., Stark, G. R. & Sen, G. C. Transcriptional induction by double-stranded RNA is mediated by interferon-stimulated response elements without activation of interferon-stimulated gene factor 3. *J. Biol. Chem.* **270**, 19624–19629 (1995). 7642650 10.1074/jbc.270.33.19624

[47] Diamond, M. S. & Farzan, M. The broad-spectrum antiviral functions of IFIT and IFITM proteins. *Nat. Rev. Immunol.* **13**, 46–57 (2013). 23237964 10.1038/nri3344 PMC3773942

[48] Fata-Hartley, C. L. & Palmenberg, A. C. Dipyridamole reversibly inhibits mengovirus RNA replication. *J. Virol.* **79**, 11062–11070 (2005). 16103157 10.1128/JVI.79.17.11062-11070.2005 PMC1193570

[49] Pulverer, J. E. et al. Temporal and spatial resolution of type I and III interferon responses in vivo. *J. Virol.* **84**, 8626–8638 (2010). 20573823 10.1128/JVI.00303-10 PMC2919002

[50] Whittemore, L. A. & Maniatis, T. Postinduction turnoff of beta-interferon gene expression. *Mol. Cell Biol.* **10**, 1329–1337 (1990). 2157136 10.1128/mcb.10.4.1329-1337.1990 PMC362234

[51] Chao, J. A., Patskovsky, Y., Almo, S. C. & Singer, R. H. Structural basis for the coevolution of a viral RNA-protein complex. *Nat. Struct. Mol. Biol.* **15**, 103–105 (2008). 18066080 10.1038/nsmb1327 PMC3152963

[52] Kamiyama, D. et al. Versatile protein tagging in cells with split fluorescent protein. *Nat. Commun.* **7**, 11046 (2016). 26988139 10.1038/ncomms11046 PMC4802074

[53] Albulescu, L., Wubbolts, R., van Kuppeveld, F. J. M. & Strating, J. R. P. M. Cholesterol shuttling is important for RNA replication of coxsackievirus B3 and encephalomyocarditis virus. *Cell Microbiol.* **17**, 1144–1156 (2015). 25645595 10.1111/cmi.12425

[54] Belov, G. A. & van Kuppeveld, F. J. M. (+)RNA viruses rewire cellular pathways to build replication organelles. *Curr. Opin. Virol.* **2**, 740–747 (2012). 23036609 10.1016/j.coviro.2012.09.006 PMC7102821

[55] Feng, Q. et al. MDA5 detects the double-stranded RNA replicative form in picornavirus-infected cells. *Cell Rep.* **2**, 1187–1196 (2012). 23142662 10.1016/j.celrep.2012.10.005 PMC7103987

[56] Melia, C. E. et al. Escaping host factor PI4KB inhibition: enterovirus genomic RNA replication in the absence of replication organelles. *Cell Rep.* **21**, 587–599 (2017). 29045829 10.1016/j.celrep.2017.09.068 PMC5656745

[57] Schuster, S., Tholen, L. E., Overheul, G. J., van Kuppeveld, F. J. M. & van Rij, R. P. Deletion of cytoplasmic double-stranded RNA sensors does not uncover viral small interfering RNA production in human cells. *mSphere* **2**, e00333-17 (2017). 28815217 10.1128/mSphere.00333-17 PMC5557678

[58] Duke, G. M. & Palmenberg, A. C. Cloning and synthesis of infectious cardiovirus RNAs containing short, discrete poly(C) tracts. *J. Virol.* **63**, 1822–1826 (1989). 2538661 10.1128/jvi.63.4.1822-1826.1989 PMC248463

[59] Yan, X., Hoek, T. A., Vale, R. D. & Tanenbaum, M. E. Dynamics of translation of single mRNA molecules in vivo. *Cell* **165**, 976–989 (2016). 27153498 10.1016/j.cell.2016.04.034 PMC4889334

[60] Lyubimova, A. et al. Single-molecule mRNA detection and counting in mammalian tissue. *Nat. Protoc.* **8**, 1743–1758 (2013). 23949380 10.1038/nprot.2013.109

[61] Gaspar, I., Wippich, F. & Ephrussi, A. Terminal deoxynucleotidyl transferase mediated production of labeled probes for single-molecule FISH or RNA capture. *Bio Protoc.* **8**, e2750 (2018). 34179277 10.21769/BioProtoc.2750 PMC8203885

[62] Stringer, C., Wang, T., Michaelos, M. & Pachitariu, M. Cellpose: a generalist algorithm for cellular segmentation. *Nat. Methods* **18**, 100–106 (2021). 33318659 10.1038/s41592-020-01018-x

[63] Ulicna, K., Vallardi, G., Charras, G. & Lowe, A. R. Automated deep lineage tree analysis using a Bayesian single cell tracking approach. *Front. Comput. Sci.* **3**, 734559 (2021).

[64] Sardá-Espinosa, A. Time-series clustering in R using the dtwclust package. *R J.*10.32614/RJ-2019-023 (2019).

