# Supplementary material for: Antiviral responses are shaped by heterogeneity in viral replication dynamics
Source: Nat Microbiol. 2023 Oct 9;8(11):2115–29. doi: 10.1038/s41564-023-01501-z (PMC10627821; doi:10.1038/s41564-023-01501-z)
Supplement: Supplementary file 1 — Reporting Summary [file 41564_2023_1501_MOESM1_ESM.pdf]

nature portfolio

Corresponding author(s): dr. Marvin Tanenbaum

Last updated by author(s): Aug 23, 2023

Reporting Summary

Nature Portfolio wishes to improve the reproducibility of the work that we publish. This form provides structure for consistency and transparency in reporting. For further information on Nature Portfolio policies, see our Editorial Policies and the Editorial Policy Checklist.

Statistics

For all statistical analyses, confirm that the following items are present in the figure legend, table legend, main text, or Methods section.

n/a	Confirmed	
☐	☒	The exact sample size (*n*) for each experimental group/condition, given as a discrete number and unit of measurement
☐	☒	A statement on whether measurements were taken from distinct samples or whether the same sample was measured repeatedly
☐	☒	The statistical test(s) used AND whether they are one- or two-sided<br>*Only common tests should be described solely by name; describe more complex techniques in the Methods section.*
☒	☐	A description of all covariates tested
☐	☒	A description of any assumptions or corrections, such as tests of normality and adjustment for multiple comparisons
☐	☒	A full description of the statistical parameters including central tendency (e.g. means) or other basic estimates (e.g. regression coefficient) AND variation (e.g. standard deviation) or associated estimates of uncertainty (e.g. confidence intervals)
☐	☒	For null hypothesis testing, the test statistic (e.g. *F*, *t*, *r*) with confidence intervals, effect sizes, degrees of freedom and *P* value noted<br>*Give P values as exact values whenever suitable.*
☒	☐	For Bayesian analysis, information on the choice of priors and Markov chain Monte Carlo settings
☒	☐	For hierarchical and complex designs, identification of the appropriate level for tests and full reporting of outcomes
☐	☒	Estimates of effect sizes (e.g. Cohen's *d*, Pearson's *r*), indicating how they were calculated

Our web collection on statistics for biologists contains articles on many of the points above.

Software and code

Policy information about availability of computer code

Data collection	All imaging experiments were performed on NIKON TI2 inverted microscope equipped with NIS elements AR software (version 5.21.03; https://www.microscope.healthcare.nikon.com/en_EU/products/software/nis-elements). For QPCR analysis BioRad Maestro (version 1.1 4.1.2433; https://www.bio-rad.com/en-nl/product/cfx-maestro-software-for-cfx-real-time-pcr-instruments?ID=OKZP7E15) was used.
Data analysis	Analysis of images was performed using FIJI (version 1.8.0_66; https://imagej.nih.gov/ij/). GraphPad PRISM 8 (version 8.2.1; www.graphpad.com) and Microsoft Excel 2019 were used for data visualization and statistical analysis. For the automated analysis of fluorescence intensity time traces and for extracting logistic fit parameters custom scripts using python code packages (including cellpose (version 0.6.5), napari (version (0.4.6) and btrack (version 0.4.0) and R packages (dtw (version1.22.3)) was used as described in the methods section. Code is available from: doi: 10.17632/8p8vy5s35b.1

For manuscripts utilizing custom algorithms or software that are central to the research but not yet described in published literature, software must be made available to editors and reviewers. We strongly encourage code deposition in a community repository (e.g. GitHub). See the Nature Portfolio guidelines for submitting code & software for further information.

Data

Policy information about availability of data

All manuscripts must include a data availability statement. This statement should provide the following information, where applicable:

- Accession codes, unique identifiers, or web links for publicly available datasets
- A description of any restrictions on data availability
- For clinical datasets or third party data, please ensure that the statement adheres to our policy

A selection of source imaging data for all figures is publicly available at Mendeley data: DOI:10.17632/8p8vy5s35b.1.

Human research participants

Policy information about studies involving human research participants and Sex and Gender in Research.

Reporting on sex and gender	n.a.
Population characteristics	n.a.
Recruitment	n.a.
Ethics oversight	n.a.

Note that full information on the approval of the study protocol must also be provided in the manuscript.

Field-specific reporting

Please select the one below that is the best fit for your research. If you are not sure, read the appropriate sections before making your selection.

☒ Life sciences ☐ Behavioural & social sciences ☐ Ecological, evolutionary & environmental sciences

For a reference copy of the document with all sections, see nature.com/documents/nr-reporting-summary-flat.pdf

Life sciences study design

All studies must disclose on these points even when the disclosure is negative.

Sample size	No a priori sample size calculations were performed. Instead, sample size was chosen based on sample size and variability observed in previous studies (Doganay, 2017; Patil, 2015; Rand, 2012). In addition sample size was determined by the number of positions that could be imaged at the given time interval. In some imaging experiments, more positions were imaged than were analyzed. In these cases a random subset of positions was analyzed (all infections within one position were analyzed). The sample size for each experiment is given in supplementary data table 2.
Data exclusions	No data was excluded
Replication	Unless otherwise stated, experiments were performed at least 3 times in biological and technical independent replicates with comparable results. The sample size and number of independent replicates for each experiment is given in supplementary data table 2. The findings in fig.4 were additionally replicated in an independent analysis: starting with the raw imaging data and a set of guidelines (described in the methods section), the analysis was performed independently by a researcher not involved in the original data analysis. The results of this replication analysis were similar to the data presented in the manuscript.
Randomization	Randomization is not relevant in this study since uninfected and infected, IFIT1+ and IFIT1-, and IFNB1+ and IFNB1- cells are all imaged in the same imaging well and these different outcomes arise during the experiment (i.e. all cells are treated the same at the start of the experiment)
Blinding	Investigators were not blinded as the experimental work was performed by the same investigator that did the analyses. In order to minimize bias in the analysis in fig. 2 and 4, viral load of infected cells was determined before assessing IFIT1 expression status.

Reporting for specific materials, systems and methods

We require information from authors about some types of materials, experimental systems and methods used in many studies. Here, indicate whether each material, system or method listed is relevant to your study. If you are not sure if a list item applies to your research, read the appropriate section before selecting a response.

Materials & experimental systems

n/a	Involved in the study	
☐	☒	Antibodies
☐	☒	Eukaryotic cell lines
☒	☐	Palaeontology and archaeology
☒	☐	Animals and other organisms
☒	☐	Clinical data
☒	☐	Dual use research of concern

Methods

n/a	Involved in the study	
☒	☐	ChIP-seq
☒	☐	Flow cytometry
☒	☐	MRI-based neuroimaging

Antibodies

Antibodies used

Monoclonal anti-dsRNA antibody (J2, Jena Bioscience, lot 18268) diluted 1:1000 (from an 1µg/µl stock solution)

Validation

A negative control (uninfected cells) was included in the experiment. The J2 dsRNA monoclonal antibody has been used extensively in the past and has been validated in previous work (among others Schönborn et al. (1991) Nucleic Acids Res.19: 2993)

Eukaryotic cell lines

Policy information about cell lines and Sex and Gender in Research

Cell line source(s)

HeLa cells provided by the lab of G. Kops, Hubrecht Institute. Cells are not commercially available
HeLa MDA5 k.o. and MAVS k.o. cells (Melia, 2017; Schuster, 2017) were previously established in the lab of F. van Kuppeveld
BHK-T7 cells provided by the lab of M. Rameix-Welti, INSERM, Université Versailles Saint-Quentin en Yvelines
HEK293T cells, Tanenbaum lab (ATCC Cat# CRL-3216)

Authentication

The cell lines used were not authenticated

Mycoplasma contamination

All cell lines were tested for mycoplasma contamination at multiple instances during the study. Results were negative

Commonly misidentified lines
(See ICLAC register)

No commonly misidentified cell line was used
