# Supplementary material for: Antiviral responses are shaped by heterogeneity in viral replication dynamics
Source: Nat Microbiol. 2023 Oct 9;8(11):2115–29. doi: 10.1038/s41564-023-01501-z (PMC10627821; doi:10.1038/s41564-023-01501-z)

SOURCE DATA

Unprocessed gel images related to Extended Data Fig. 3B

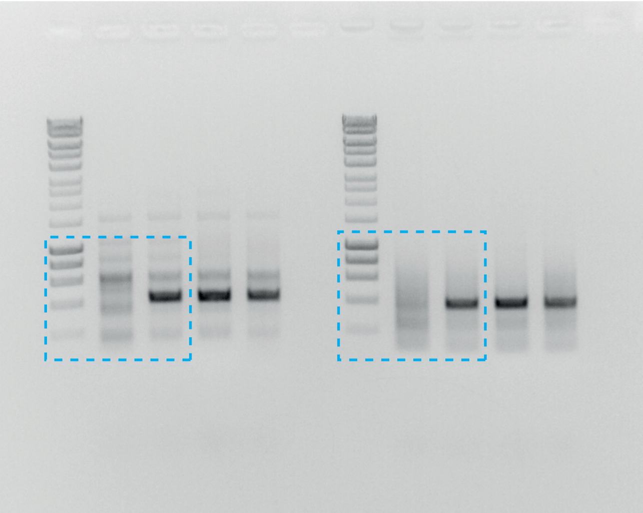

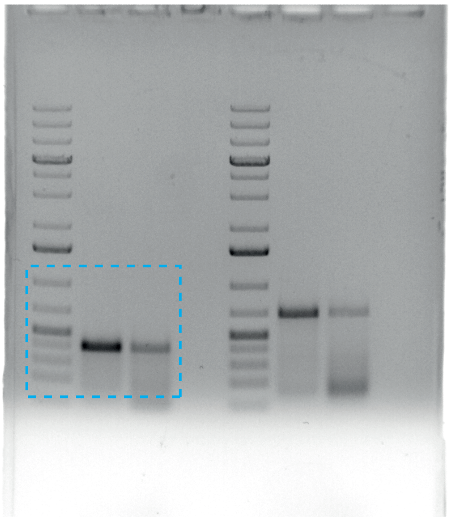

Supplement: Supplementary file 6 — Unprocessed gel images. [file 41564_2023_1501_MOESM6_ESM.pdf]
